# A scoping review of continuous quality improvement in healthcare system: conceptualization, models and tools, barriers and facilitators, and impact

**DOI:** 10.1186/s12913-024-10828-0

**Published:** 2024-04-19

**Authors:** Aklilu Endalamaw, Resham B Khatri, Tesfaye Setegn Mengistu, Daniel Erku, Eskinder Wolka, Anteneh Zewdie, Yibeltal Assefa

**Affiliations:** 1 School of Public Health, The University of Queensland, Brisbane, Australia; 2 College of Medicine and Health Sciences, Bahir Dar University, Bahir Dar, Ethiopia; 3 Health Social Science and Development Research Institute, Kathmandu, Nepal; 4 Centre for Applied Health Economics, School of Medicine, Grifth University, Brisbane, Australia; 5 Menzies Health Institute Queensland, Grifth University, Brisbane, Australia; 6 International Institute for Primary Health Care in Ethiopia, Addis Ababa, Ethiopia

**Keywords:** Continuous quality improvement, Quality of Care

## Abstract

**Background:**

The growing adoption of continuous quality improvement (CQI) initiatives in healthcare has generated a surge in research interest to gain a deeper understanding of CQI. However, comprehensive evidence regarding the diverse facets of CQI in healthcare has been limited. Our review sought to comprehensively grasp the conceptualization and principles of CQI, explore existing models and tools, analyze barriers and facilitators, and investigate its overall impacts.

**Methods:**

This qualitative scoping review was conducted using Arksey and O’Malley’s methodological framework. We searched articles in PubMed, Web of Science, Scopus, and EMBASE databases. In addition, we accessed articles from Google Scholar. We used mixed-method analysis, including qualitative content analysis and quantitative descriptive for quantitative findings to summarize findings and PRISMA extension for scoping reviews (PRISMA-ScR) framework to report the overall works.

**Results:**

A total of 87 articles, which covered 14 CQI models, were included in the review. While 19 tools were used for CQI models and initiatives, Plan-Do-Study/Check-Act cycle was the commonly employed model to understand the CQI implementation process. The main reported purposes of using CQI, as its positive impact, are to improve the structure of the health system (e.g., leadership, health workforce, health technology use, supplies, and costs), enhance healthcare delivery processes and outputs (e.g., care coordination and linkages, satisfaction, accessibility, continuity of care, safety, and efficiency), and improve treatment outcome (reduce morbidity and mortality). The implementation of CQI is not without challenges. There are cultural (i.e., resistance/reluctance to quality-focused culture and fear of blame or punishment), technical, structural (related to organizational structure, processes, and systems), and strategic (inadequate planning and inappropriate goals) related barriers that were commonly reported during the implementation of CQI.

**Conclusions:**

Implementing CQI initiatives necessitates thoroughly comprehending key principles such as teamwork and timeline. To effectively address challenges, it’s crucial to identify obstacles and implement optimal interventions proactively. Healthcare professionals and leaders need to be mentally equipped and cognizant of the significant role CQI initiatives play in achieving purposes for quality of care.

**Supplementary Information:**

The online version contains supplementary material available at 10.1186/s12913-024-10828-0.

## Background

Continuous quality improvement (CQI) initiative is a crucial initiative aimed at enhancing quality in the health system that has gradually been adopted in the healthcare industry. In the early 20th century, Shewhart laid the foundation for quality improvement by describing three essential steps for process improvement: specification, production, and inspection [1, 2]. Then, Deming expanded Shewhart’s three-step model into ‘plan, do, study/check, and act’ (PDSA or PDCA) cycle, which was applied to management practices in Japan in the 1950s [3] and was gradually translated into the health system. In 1991, Kuperman applied a CQI approach to healthcare, comprising selecting a process to be improved, assembling a team of expert clinicians that understands the process and the outcomes, determining key steps in the process and expected outcomes, collecting data that measure the key process steps and outcomes, and providing data feedback to the practitioners [4]. These philosophies have served as the baseline for the foundation of principles for continuous improvement [5].

Continuous quality improvement fosters a culture of continuous learning, innovation, and improvement. It encourages proactive identification and resolution of problems, promotes employee engagement and empowerment, encourages trust and respect, and aims for better quality of care [6, 7]. These characteristics drive the interaction of CQI with other quality improvement projects, such as quality assurance and total quality management [8]. Quality assurance primarily focuses on identifying deviations or errors through inspections, audits, and formal reviews, often settling for what is considered ‘good enough’, rather than pursuing the highest possible standards [9, 10], while total quality management is implemented as the management philosophy and system to improve all aspects of an organization continuously [11].

Continuous quality improvement has been implemented to provide quality care. However, providing effective healthcare is a complicated and complex task in achieving the desired health outcomes and the overall well-being of individuals and populations. It necessitates tackling issues, including access, patient safety, medical advances, care coordination, patient-centered care, and quality monitoring [12, 13], rooted long ago. It is assumed that the history of quality improvement in healthcare started in 1854 when Florence Nightingale introduced quality improvement documentation [14]. Over the passing decades, Donabedian introduced structure, processes, and outcomes as quality of care components in 1966 [15]. More comprehensively, the Institute of Medicine in the United States of America (USA) has identified effectiveness, efficiency, equity, patient-centredness, safety, and timeliness as the components of quality of care [16]. Moreover, quality of care has recently been considered an integral part of universal health coverage (UHC) [17], which requires initiatives to mobilise essential inputs [18].

While the overall objective of CQI in health system is to enhance the quality of care, it is important to note that the purposes and principles of CQI can vary across different contexts [19, 20]. This variation has sparked growing research interest. For instance, a review of CQI approaches for capacity building addressed its role in health workforce development [21]. Another systematic review, based on random-controlled design studies, assessed the effectiveness of CQI using training as an intervention and the PDSA model [22]. As a research gap, the former review was not directly related to the comprehensive elements of quality of care, while the latter focused solely on the impact of training using the PDSA model, among other potential models. Additionally, a review conducted in 2015 aimed to identify barriers and facilitators of CQI in Canadian contexts [23]. However, all these reviews presented different perspectives and investigated distinct outcomes. This suggests that there is still much to explore in terms of comprehensively understanding the various aspects of CQI initiatives in healthcare.

As a result, we conducted a scoping review to address several aspects of CQI. Scoping reviews serve as a valuable tool for systematically mapping the existing literature on a specific topic. They are instrumental when dealing with heterogeneous or complex bodies of research. Scoping reviews provide a comprehensive overview by summarizing and disseminating findings across multiple studies, even when evidence varies significantly [24]. In our specific scoping review, we included various types of literature, including systematic reviews, to enhance our understanding of CQI.

This scoping review examined how CQI is conceptualized and measured and investigated models and tools for its application while identifying implementation challenges and facilitators. It also analyzed the purposes and impact of CQI on the health systems, providing valuable insights for enhancing healthcare quality.

## Methods

### Protocol registration and results reporting

Protocol registration for this scoping review was not conducted. Arksey and O’Malley’s methodological framework was utilized to conduct this scoping review [25]. The scoping review procedures start by defining the research questions, identifying relevant literature, selecting articles, extracting data, and summarizing the results. The review findings are reported using the PRISMA extension for a scoping review (PRISMA-ScR) [26]. McGowan and colleagues also advised researchers to report findings from scoping reviews using PRISMA-ScR [27].

### Defining the research problems

This review aims to comprehensively explore the conceptualization, models, tools, barriers, facilitators, and impacts of CQI within the healthcare system worldwide. Specifically, we address the following research questions: (1) How has CQI been defined across various contexts? (2) What are the diverse approaches to implementing CQI in healthcare settings? (3) Which tools are commonly employed for CQI implementation ? (4) What barriers hinder and facilitators support successful CQI initiatives? and (5) What effects CQI initiatives have on the overall care quality?

### Information source and search strategy

We conducted the search in PubMed, Web of Science, Scopus, and EMBASE databases, and the Google Scholar search engine. The search terms were selected based on three main distinct concepts. One group was CQI-related terms. The second group included terms related to the purpose for which CQI has been implemented, and the third group included processes and impact. These terms were selected based on the Donabedian framework of structure, process, and outcome [28]. Additionally, the detailed keywords were recruited from the primary health framework, which has described lists of dimensions under process, output, outcome, and health system goals of any intervention for health [29]. The detailed search strategy is presented in the Supplementary file 1 (Search strategy). The search for articles was initiated on August 12, 2023, and the last search was conducted on September 01, 2023.

### Eligibility criteria and article selection

Based on the scoping review’s population, concept, and context frameworks [30], the population included any patients or clients. Additionally, the concepts explored in the review encompassed definitions, implementation, models, tools, barriers, facilitators, and impacts of CQI. Furthermore, the review considered contexts at any level of health systems. We included articles if they reported results of qualitative or quantitative empirical study, case studies, analytic or descriptive synthesis, any review, and other written documents, were published in peer-reviewed journals, and were designed to address at least one of the identified research questions or one of the identified implementation outcomes or their synonymous taxonomy as described in the search strategy. Based on additional contexts, we included articles published in English without geographic and time limitations. We excluded articles with abstracts only, conference abstracts, letters to editors, commentators, and corrections.

We exported all citations to EndNote x20 to remove duplicates and screen relevant articles. The article selection process includes automatic duplicate removal by using EndNote x20, unmatched title and abstract removal, citation and abstract-only materials removal, and full-text assessment. The article selection process was mainly conducted by the first author (AE) and reported to the team during the weekly meetings. The first author encountered papers that caused confusion regarding whether to include or exclude them and discussed them with the last author (YA). Then, decisions were ultimately made. Whenever disagreements happened, they were resolved by discussion and reconsideration of the review questions in relation to the written documents of the article. Further statistical analysis, such as calculating Kappa, was not performed to determine article inclusion or exclusion.

### Data extraction and data items

We extracted first author, publication year, country, settings, health problem, the purpose of the study, study design, types of intervention if applicable, CQI approaches/steps if applicable, CQI tools and procedures if applicable, and main findings using a customized Microsoft Excel form.

### Summarizing and reporting the results

The main findings were summarized and described based on the main themes, including concepts under conceptualizing, principles, teams, timelines, models, tools, barriers, facilitators, and impacts of CQI. Results-based convergent synthesis, achieved through mixed-method analysis, involved content analysis to identify the thematic presentation of findings. Additionally, a narrative description was used for quantitative findings, aligning them with the appropriate theme. The authors meticulously reviewed the primary findings from each included material and contextualized these findings concerning the main themes1. This approach provides a comprehensive understanding of complex interventions and health systems, acknowledging quantitative and qualitative evidence.

## Results

### Search results

**Fig. 1:**
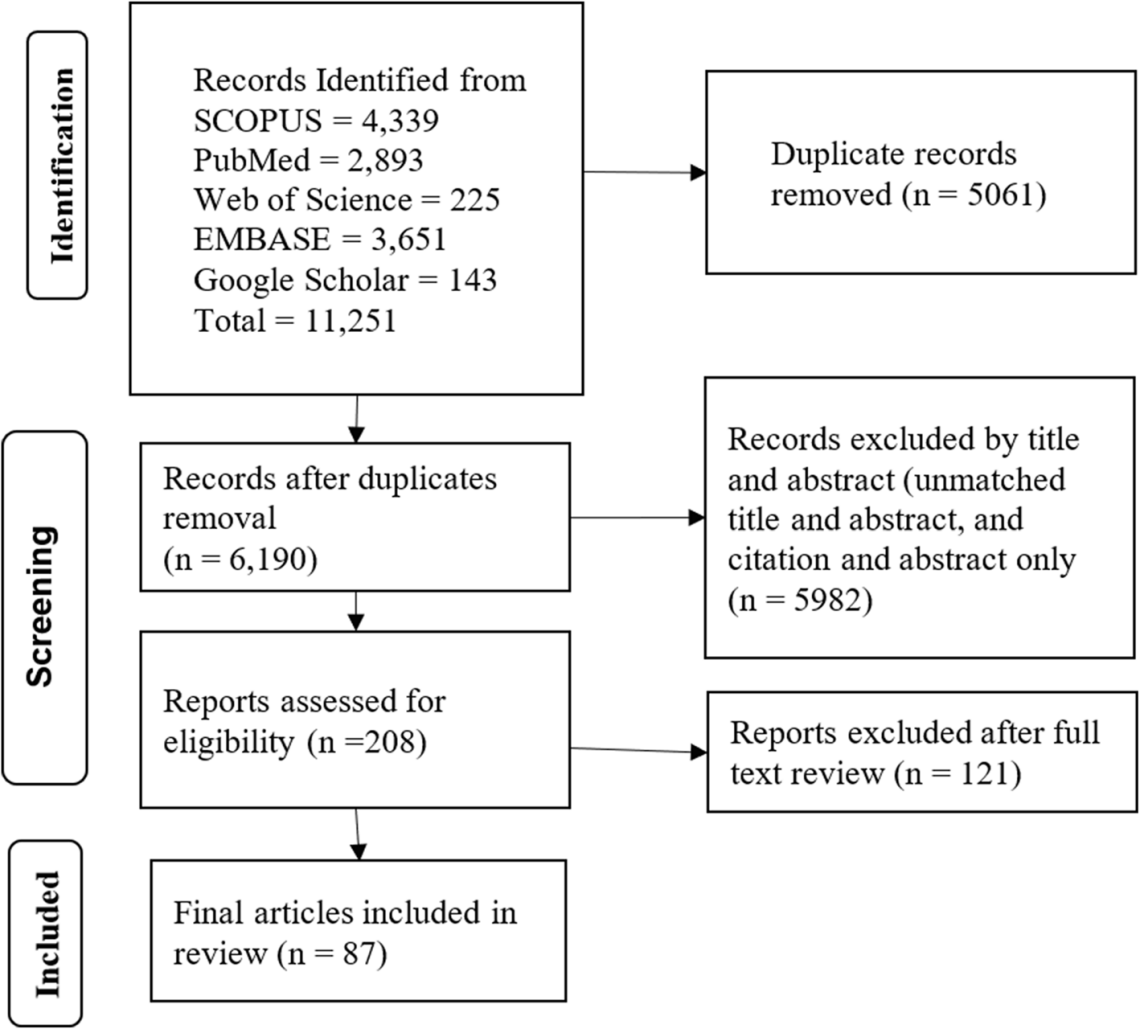
Article selection process

A total of 11,251 documents were identified from various databases: SCOPUS (*n* = 4,339), PubMed (*n* = 2,893), Web of Science (*n* = 225), EMBASE (*n* = 3,651), and Google Scholar (*n* = 143). After removing duplicates (*n* = 5,061), 6,190 articles were evaluated by title and abstract. Subsequently, 208 articles were assessed for full-text eligibility. Following the eligibility criteria, 121 articles were excluded, leaving 87 included in the current review (Fig. 1).

### Operationalizing continuous quality improvement

Continuous Quality Improvement (CQI) is operationalized as a cyclic process that requires commitment to implementation, teamwork, time allocation, and celebrating successes and failures.

- CQI is a cyclic ongoing process that is followed reflexive, analytical and iterative steps, including identifying gaps, generating data, developing and implementing action plans, evaluating performance, providing feedback to implementers and leaders, and proposing necessary adjustments [31–38].
- CQI requires committing to the philosophy, involving continuous improvement [19, 38], establishing a mission statement [37], and understanding quality definition [19].
- CQI involves a wide range of patient-oriented measures and performance indicators, specifically satisfying internal and external customers, developing quality assurance, adopting common quality measures, and selecting process measures [8, 19, 35–37, 39, 40].
- CQI requires celebrating success and failure without personalization, leading each team member to develop error-free attitudes [19]. Success and failure are related to underlying organizational processes and systems as causes of failure rather than blaming individuals [8] because CQI is process-focused based on collaborative, data-driven, responsive, rigorous and problem-solving statistical analysis [8, 19, 38]. Furthermore, a gap or failure opens another opportunity for establishing a data-driven learning organization [41].
- CQI cannot be implemented without a CQI team [8, 19, 37, 39, 42–46]. A CQI team comprises individuals from various disciplines, often comprising a team leader, a subject matter expert (physician or other healthcare provider), a data analyst, a facilitator, frontline staff, and stakeholders [39, 43, 47–49]. It is also important to note that inviting stakeholders or partners as part of the CQI support intervention is crucial [19, 38, 48].
- The timeline is another distinct feature of CQI because the results of CQI vary based on the implementation duration of each cycle [35]. There is no specific time limit for CQI implementation, although there is a general consensus that a cycle of CQI should be relatively short [35]. For instance, a CQI implementation took 2 months [42], 4 months [50], 9 months [51, 52], 12 months [53–55], and one year and 5 months [49] duration to achieve the desired positive outcome, while bi-weekly [47] and monthly data reviews and analyses [44, 48, 56], and activities over 3 months [57] have also resulted in a positive outcome.

### Continuous quality improvement models and tools

There have been several models are utilized. The Plan-Do-Study/Check-Act cycle is a stepwise process involving project initiation, situation analysis, root cause identification, solution generation and selection, implementation, result evaluation, standardization, and future planning [7, 36, 37, 45, 47–51, 53, 56–70]. The FOCUS-PDCA cycle enhances the PDCA process by adding steps to find and improve a process (F), organize a knowledgeable team (O), clarify the process (C), understand variations (U), and select improvements (S) [55, 71–73]. The FADE cycle involves identifying a problem (Focus), understanding it through data analysis (Analyze), devising solutions (Develop), and implementing the plan (Execute) [74]. The Logic Framework involves brainstorming to identify improvement areas, conducting root cause analysis to develop a problem tree, logically reasoning to create an objective tree, formulating the framework, and executing improvement projects [75]. Breakthrough series approach requires CQI teams to meet in quarterly collaborative learning sessions, share learning experiences, and continue discussion by telephone and cross-site visits to strengthen learning and idea exchange [47]. Another CQI model is the Lean approach, which has been conducted with Kaizen principles [52], 5 S principles, and the Six Sigma model. The 5 S (Sort, Set/Straighten, Shine, Standardize, Sustain) systematically organises and improves the workplace, focusing on sorting, setting order, shining, standardizing, and sustaining the improvement [54, 76]. Kaizen principles guide CQI by advocating for continuous improvement, valuing all ideas, solving problems, focusing on practical, low-cost improvements, using data to drive change, acknowledging process defects, reducing variability and waste, recognizing every interaction as a customer-supplier relationship, empowering workers, responding to all ideas, and maintaining a disciplined workplace [77]. Lean Six Sigma, a CQI model, applies the DMAIC methodology, which involves defining (D) and measuring the problem (M), analyzing root causes (A), improving by finding solutions (I), and controlling by assessing process stability (C) [78, 79]. The 5 C-cyclic model (consultation, collection, consideration, collaboration, and celebration), the first CQI framework for volunteer dental services in Aboriginal communities, ensures quality care based on community needs [80]. One study used meetings involving activities such as reviewing objectives, assigning roles, discussing the agenda, completing tasks, retaining key outputs, planning future steps, and evaluating the meeting’s effectiveness [81].

Various tools are involved in the implementation or evaluation of CQI initiatives: checklists [53, 82], flowcharts [81–83], cause-and-effect diagrams (fishbone or Ishikawa diagrams) [60, 62, 79, 81, 82], fuzzy Pareto diagram [82], process maps [60], time series charts [48], why-why analysis [79], affinity diagrams and multivoting [81], and run chart [47, 48, 51, 60, 84], and others mentioned in the table (Table 1).

**Table 1:** Different CQI tools and their purpose in CQI initiative implementation

Tools	Purpose of CQI initiative implementation	Models used
Checklist	Immunization program [44], maternal and child health [76], and healthcare financing [73]	PDCA
Flowcharts	Healthcare costs [73], frequency of episiotomy procedures [74], and immunization rate [72]	Lean Six Sigma, FOCUS-PDCA cycle, Meeting
Cause-and-effect diagrams (fishbone or Ishikawa diagrams)	Healthcare financing [73], reducing overcrowding and improving the patient discharge process [70], ANC HIV testing [51], preventing infection post caesarean section surgery [53], increasing immunization rate [72], and length of stay [15]	Lean Six Sigma, DMAIC; Meeting, PDSA, Baldridge criteria, FADE, Logical framework
Pareto diagram	Healthcare financing [73] and length of stay hospitals [15]	
Process maps	ANC HIV testing [51]	PDSA
Time series charts	VMMC [39]	PDSA
Why-Why diagram	Reduce overcrowding and improving patient discharge process [70]	Lean Six Sigma, DMAIC
Affinity diagrams	Increasing immunization rate [72]	Meeting
Multivoting	Increasing immunization rate [72]	Meeting and PDCA
Run chart	HIV/AIDS responses (HIV testing, VMMC, PMTCT care) [38, 39, 42, 51], and continuity of child care [75]	PDSA/PDCA
Table	Diabetic care [31]	
Pie charts	Diabetic care [31]	
Histograms	Diabetic care [31]	
Boxplots	Diabetic care [31]	
Star plots	Diabetic care [31]	
Variability graph	Diabetic care [31]	
P-charts	Maternal care [77]	
Chart sticker	Pressure ulcer care [61]	PDCA
Control chart	Patient satisfaction and overall quality [78] and radiotherapy care [79]	PDCA

### Barriers and facilitators of continuous quality improvement implementation

Implementing CQI initiatives is determined by various barriers and facilitators, which can be thematized into four dimensions. These dimensions are cultural, technical, structural, and strategic dimensions.

Continuous quality improvement initiatives face various cultural, strategic, technical, and structural barriers. Cultural dimension barriers involve resistance to change (e.g., not accepting online technology), lack of quality-focused culture, staff reporting apprehensiveness, and fear of blame or punishment [36, 41, 85, 86]. The technical dimension barriers of CQI can include various factors that hinder the effective implementation and execution of CQI processes [36, 86–89]. Structural dimension barriers of CQI arise from the organization structure, process, and systems that can impede the effective implementation and sustainability of CQI [36, 85–88]. Strategic dimension barriers are, for example, the inability to select proper CQI goals and failure to integrate CQI into organizational planning and goals [36, 85–88, 90].

Facilitators are also grouped to cultural, structural, technical, and strategic dimensions to provide solutions to CQI barriers. Cultural challenges were addressed by developing a group culture to CQI and other rewards [39, 41, 80, 85–87, 90–92]. Technical facilitators are pivotal to improving technical barriers [39, 42, 53, 69, 86, 90, 91]. Structural-related facilitators are related to improving communication, infrastructure, and systems [86, 92, 93]. Strategic dimension facilitators include strengthening leadership and improving decision-making skills [43, 53, 67, 86, 87, 92, 94, 95] (Table 2).

**Table 2:** Summary of barriers and facilitators to CQI implementation

Dimensions	Barriers	Facilitators
Cultural dimension	• Physician decline membership of CQI [27] • Non-involvement of all pharmacy staff [76] • Staff resistance to change [76] • Absence of celebration or rewards for achievement [77] • Hierarchical culture [32, 77] • Rational culture [32, 77] • Staffs’ reluctance to report errors [76]	• Development of a culture and group culture to CQI [32, 77] • Perception of feasibility, confidentiality, receptive attitudes, a sense of ownership, and perceptions of positive impacts [82] • Managers commitment for quality-related event reporting and learning [76] • Inviting physicians to join the quality journey [30] • Involving patients, families, leaders, and staffs [83] • Gather all personnel to collaborate for a common goal [71, 77] • Teamwork [77, 78] • Rewarding and celebrating success [71, 81]
Technical dimension	• Inadequate capitalization of project and insufficient support for CQI facilitators and data entry managers [27] • Immature electronic medical records or poor information systems [27] • Lack of training opportunities and skills [77–79] • Difficulty of finding codes for conditions and procedures [80] • The high rate of non-codable items [80] • The lack of recommended measures [80]	• Continued seminar, education, and training [30, 33, 44, 60, 77, 81, 82] • Assessing a limited but essential number of quality indicators [82] • Data quality and availability [77] • Continuous and reliable information, including measurement, about test and current practice [83] • Developing a manual-online hybrid reporting system [76]
Structural dimension	• Weak or absence of physician-to-physician cooperation and synergies [27] • Changed staff relationship [76] • Lack of mechanisms for disseminating knowledge [77] • Limited use of communication mechanisms [77] • Staff shortages and turnover [78] • Insufficient staffing [79]	• Effective forums of communication [77] • An infrastructure based on improvement in knowledge [83] • Learning systems and sustainability systems [83] • Improving information systems [84] • Adopting systematic problem-solving approaches [84]
Strategic dimension	• Inability to select proper goals of CQI [27] • Poor planning [79] • Failure to integrate CQI into organizational planning and goals [27] • Unalignment of goals and priorities of leadership and management [77] • Fragmentation of quality assurance policies [78] • Inadequate financial or other positive reinforcement to staffs [27] • Lack of support [81] • Resource inadequacy [77] • Time constraint [76, 77] • work overload [77].	• Strengthened leadership [77, 78] • CQI-based mentoring [85] • Periodic monitoring, supportive supervision, and coaching [34, 44, 78, 83, 86] • Participation, empowerment, and accountability [58] • Involving all stakeholders in decision-making [77, 78] • A provider-payer partnership [55] • Compensating staff for after-hours meetings on CQI [76] • The adoption of a formative approach to CQI implementation [82].

### Impact of continuous quality improvement

Continuous quality improvement initiatives can significantly impact the quality of healthcare in a wide range of health areas, focusing on improving structure, the health service delivery process and improving client wellbeing and reducing mortality.

### Structure components

These are health leadership, financing, workforce, technology, and equipment and supplies. CQI has improved planning, monitoring and evaluation [48, 53], and leadership and planning [48], indicating improvement in leadership perspectives. Implementing CQI in primary health care (PHC) settings has shown potential for maintaining or reducing operation costs [67]. Findings from another study indicate that the costs associated with implementing CQI interventions per facility ranged from approximately $2,000 to $10,500 per year, with an average cost of approximately $10 to $60 per admitted client [57]. However, based on model predictions, the average cost savings after implementing CQI were estimated to be $5430 [31]. CQI can also be applied to health workforce development [32]. CQI in the institutional system improved medical education [66, 96, 97], human resources management [53], motivated staffs [76], and increased staff health awareness [69], while concerns raised about CQI impartiality, independence, and public accountability [96]. Regarding health technology, CQI also improved registration and documentation [48, 53, 98]. Furthermore, the CQI initiatives increased cleanliness [54] and improved logistics, supplies, and equipment [48, 53, 68].

### Process and output components

The process component focuses on the activities and actions involved in delivering healthcare services.

#### Service delivery

CQI interventions improved service delivery [53, 56, 99], particularly a significant 18% increase in the overall quality of service performance [48], improved patient counselling, adherence to appropriate procedures, and infection prevention [48, 68], and optimised workflow [52].

#### Coordination and collaboration

CQI initiatives improved coordination and collaboration through collecting and analysing data, onsite technical support, training, supportive supervision [53] and facilitating linkages between work processes and a quality control group [65].

#### Patient satisfaction

The CQI initiatives increased patient satisfaction and improved quality of life by optimizing care quality management, improving the quality of clinical nursing, reducing nursing defects and enhancing the wellbeing of clients [54, 76, 100], although CQI was not associated with changes in adolescent and young adults’ satisfaction [51].

#### Safety

CQI initiatives reduced medication error reports from 16 to 6 [101], and it significantly reduced the administration of inappropriate prophylactic antibiotics [44], decreased errors in inpatient care [52], decreased the overall episiotomy rate from 44.5 to 33.3% [83], reduced the overall incidence of unplanned endotracheal extubation [102], improving appropriate use of computed tomography angiography [103], and appropriate diagnosis and treatment selection [47].

#### Continuity of care

CQI initiatives effectively improve continuity of care by improving client and physician interaction. For instance, provider continuity levels showed a 64% increase [55]. Modifying electronic medical record templates, scheduling, staff and parental education, standardization of work processes, and birth to 1-year age-specific incentives in post-natal follow-up care increased continuity of care to 74% in 2018 compared to baseline 13% in 2012 [84].

#### Efficiency

The CQI initiative yielded enhanced efficiency in the cardiac catheterization laboratory, as evidenced by improved punctuality in procedure starts and increased efficiency in manual sheath-pulls inside [78].

#### Accessibility

CQI initiatives were effective in improving accessibility in terms of increasing service coverage and utilization rate. For instance, screening for cigarettes, nutrition counselling, folate prescription, maternal care, immunization coverage [53, 81, 104, 105], reducing the percentage of non-attending patients to surgery to 0.9% from the baseline 3.9% [43], increasing Chlamydia screening rates from 29 to 60% [45], increasing HIV care continuum coverage [51, 59, 60], increasing in the uptake of postpartum long-acting reversible contraceptive use from 6.9% at the baseline to 25.4% [42], increasing post-caesarean section prophylaxis from 36 to 89% [62], a 31% increase of kangaroo care practice [50], and increased follow-up [65]. Similarly, the QI intervention increased the quality of antenatal care by 29.3%, correct partograph use by 51.7%, and correct active third-stage labour management, a 19.6% improvement from the baseline, but not significantly associated with improvement in contraceptive service uptake [61].

#### Timely access

CQI interventions improved the time care provision [52], and reduced waiting time [62, 74, 76, 106]. For instance, the discharge process waiting time in the emergency department decreased from 76 min to 22 min [79]. It also reduced mean postprocedural length of stay from 2.8 days to 2.0 days [31].

#### Acceptability

Acceptability of CQI by healthcare providers was satisfactory. For instance, 88% of the faculty, 64% of the residents, and 82% of the staff believed CQI to be useful in the healthcare clinic [107].

### Outcome components

#### Morbidity and mortality

**Fig. 2:**
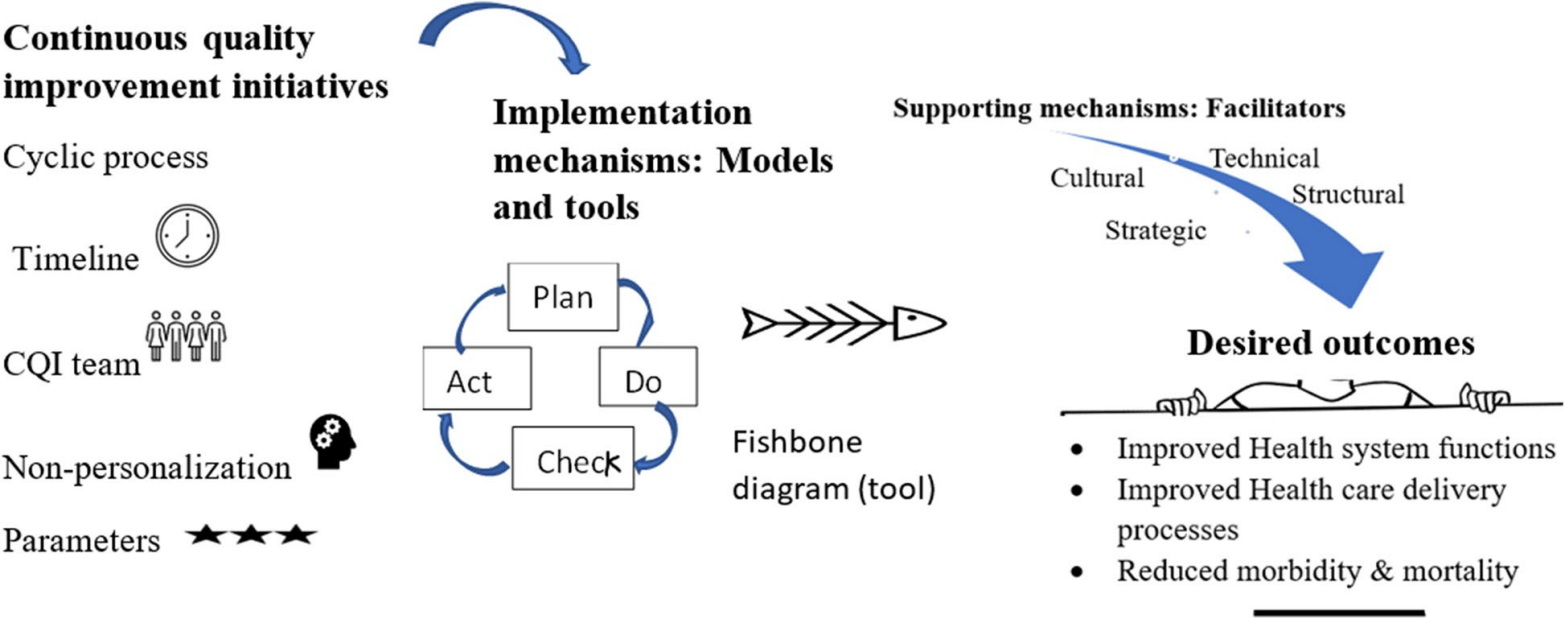
The overall mechanisms of continuous quality improvement implementation

CQI efforts have demonstrated better management outcomes among diabetic patients [40], patients with oral mucositis [71], and anaemic patients [72]. It has also reduced infection rate in post-caesarean Sect. [62], reduced post-peritoneal dialysis peritonitis [49, 108], and prevented pressure ulcers [70]. It is explained by peritonitis incidence from once every 40.1 patient months at baseline to once every 70.8 patient months after CQI [49] and a 63% reduction in pressure ulcer prevalence within 2 years from 2008 to 2010 [70]. Furthermore, CQI initiatives significantly reduced in-hospital deaths [31] and increased patient survival rates [108]. Figure 2 displays the overall process of the CQI implementations.

## Discussion

In this review, we examined the fundamental concepts and principles underlying CQI, the factors that either hinder or assist in its successful application and implementation, and the purpose of CQI in enhancing quality of care across various health issues.

Our findings have brought attention to the application and implementation of CQI, emphasizing its underlying concepts and principles, as evident in the existing literature [31–36, 39, 40, 43, 45, 46]. Continuous quality improvement has shared with the principles of continuous improvement, such as a customer-driven focus, effective leadership, active participation of individuals, a process-oriented approach, systematic implementation, emphasis on design improvement and prevention, evidence-based decision-making, and fostering partnership [5]. Moreover, Deming’s 14 principles laid the foundation for CQI principles [109]. These principles have been adapted and put into practice in various ways: ten [19] and five [38] principles in hospitals, five principles for capacity building [38], and two principles for medication error prevention [41]. As a principle, the application of CQI can be process-focused [8, 19] or impact-focused [38]. Impact-focused CQI focuses on achieving specific outcomes or impacts, whereas process-focused CQI prioritizes and improves the underlying processes and systems. These principles complement each other and can be utilized based on the objectives of quality improvement initiatives in healthcare settings. Overall, CQI is an ongoing educational process that requires top management’s involvement, demands coordination across departments, encourages the incorporation of views beyond clinical area, and provides non-judgemental evidence based on objective data [110].

The current review recognized that it was not easy to implement CQI. It requires reasonable utilization of various models and tools. The application of each tool can be varied based on the studied health problem and the purpose of CQI initiative [111], varied in context, content, structure, and usability [112]. Additionally, overcoming the cultural, technical, structural, and strategic-related barriers. These barriers have emerged from clinical staff, managers, and health systems perspectives. Of the cultural obstacles, staff non-involvement, resistance to change, and reluctance to report error were staff-related. In contrast, others, such as the absence of celebration for success and hierarchical and rational culture, may require staff and manager involvement. Staff members may exhibit reluctance in reporting errors due to various cultural factors, including lack of trust, hierarchical structures, fear of retribution, and a blame-oriented culture. These challenges pose obstacles to implementing standardized CQI practices, as observed, for instance, in community pharmacy settings [85]. The hierarchical culture, characterized by clearly defined levels of power, authority, and decision-making, posed challenges to implementing CQI initiatives in public health [41, 86]. Although rational culture, a type of organizational culture, emphasizes logical thinking and rational decision-making, it can also create challenges for CQI implementation [41, 86] because hierarchical and rational cultures, which emphasize bureaucratic norms and narrow definitions of achievement, were found to act as barriers to the implementation of CQI [86]. These could be solved by developing a shared mindset and collective commitment, establishing a shared purpose, developing group norms, and cultivating psychological preparedness among staff, managers, and clients to implement and sustain CQI initiatives. Furthermore, reversing cultural-related barriers necessitates cultural-related solutions: development of a culture and group culture to CQI [41, 86], positive comprehensive perception [91], commitment [85], involving patients, families, leaders, and staff [39, 92], collaborating for a common goal [80, 86], effective teamwork [86, 87], and rewarding and celebrating successes [80, 90].

The technical dimension barriers of CQI can include inadequate capitalization of a project and insufficient support for CQI facilitators and data entry managers [36], immature electronic medical records or poor information systems [36, 86], and the lack of training and skills [86–88]. These challenges may cause the CQI team to rely on outdated information and technologies. The presence of barriers on the technical dimension may challenge the solid foundation of CQI expertise among staff, the ability to recognize opportunities for improvement, a comprehensive understanding of how services are produced and delivered, and routine use of expertise in daily work. Addressing these technical barriers requires knowledge creation activities (training, seminar, and education) [39, 42, 53, 69, 86, 90, 91], availability of quality data [86], reliable information [92], and a manual-online hybrid reporting system [85].

Structural dimension barriers of CQI include inadequate communication channels and lack of standardized process, specifically weak physician-to-physician synergies [36], lack of mechanisms for disseminating knowledge and limited use of communication mechanisms [86]. Lack of communication mechanism endangers sharing ideas and feedback among CQI teams, leading to misunderstandings, limited participation and misinterpretations, and a lack of learning [113]. Knowledge translation facilitates the co-production of research, subsequent diffusion of knowledge, and the developing stakeholder’s capacity and skills [114]. Thus, the absence of a knowledge translation mechanism may cause missed opportunities for learning, inefficient problem-solving, and limited creativity. To overcome these challenges, organizations should establish effective communication and information systems [86, 93] and learning systems [92]. Though CQI and knowledge translation have interacted with each other, it is essential to recognize that they are distinct. CQI focuses on process improvement within health care systems, aiming to optimize existing processes, reduce errors, and enhance efficiency.

In contrast, knowledge translation bridges the gap between research evidence and clinical practice, translating research findings into actionable knowledge for practitioners. While both CQI and knowledge translation aim to enhance health care quality and patient outcomes, they employ different strategies: CQI utilizes tools like Plan-Do-Study-Act cycles and statistical process control, while knowledge translation involves knowledge synthesis and dissemination. Additionally, knowledge translation can also serve as a strategy to enhance CQI. Both concepts share the same principle: continuous improvement is essential for both. Therefore, effective strategies on the structural dimension may build efficient and effective steering councils, information systems, and structures to diffuse learning throughout the organization.

Strategic factors, such as goals, planning, funds, and resources, determine the overall purpose of CQI initiatives. Specific barriers were improper goals and poor planning [36, 86, 88], fragmentation of quality assurance policies [87], inadequate reinforcement to staff [36, 90], time constraints [85, 86], resource inadequacy [86], and work overload [86]. These barriers can be addressed through strengthening leadership [86, 87], CQI-based mentoring [94], periodic monitoring, supportive supervision and coaching [43, 53, 87, 92, 95], participation, empowerment, and accountability [67], involving all stakeholders in decision-making [86, 87], a provider-payer partnership [64], and compensating staff for after-hours meetings on CQI [85]. The strategic dimension, characterized by a strategic plan and integrated CQI efforts, is devoted to processes that are central to achieving strategic priorities. Roles and responsibilities are defined in terms of integrated strategic and quality-related goals [115].

The utmost goal of CQI has been to improve the quality of care, which is usually revealed by structure, process, and outcome. After resolving challenges and effectively using tools and running models, the goal of CQI reflects the ultimate reason and purpose of its implementation. First, effectively implemented CQI initiatives can improve leadership, health financing, health workforce development, health information technology, and availability of supplies as the building blocks of a health system [31, 48, 53, 68, 98]. Second, effectively implemented CQI initiatives improved care delivery process (counselling, adherence with standards, coordination, collaboration, and linkages) [48, 53, 65, 68]. Third, the CQI can improve outputs of healthcare delivery, such as satisfaction, accessibility (timely access, utilization), continuity of care, safety, efficiency, and acceptability [52, 54, 55, 76, 78]. Finally, the effectiveness of the CQI initiatives has been tested in enhancing responses related to key aspects of the HIV response, maternal and child health, non-communicable disease control, and others (e.g., surgery and peritonitis). However, it is worth noting that CQI initiative has not always been effective. For instance, CQI using a two- to nine-times audit cycle model through systems assessment tools did not bring significant change to increase syphilis testing performance [116]. This study was conducted within the context of Aboriginal and Torres Strait Islander people’s primary health care settings. Notably, ‘the clinics may not have consistently prioritized syphilis testing performance in their improvement strategies, as facilitated by the CQI program’ [116]. Additionally, by applying CQI-based mentoring, uptake of facility-based interventions was not significantly improved, though it was effective in increasing community health worker visits during pregnancy and the postnatal period, knowledge about maternal and child health and exclusive breastfeeding practice, and HIV disclosure status [117]. The study conducted in South Africa revealed no significant association between the coverage of facility-based interventions and Continuous Quality Improvement (CQI) implementation. This lack of association was attributed to the already high antenatal and postnatal attendance rates in both control and intervention groups at baseline, leaving little room for improvement. Additionally, the coverage of HIV interventions remained consistently high throughout the study period [117].

Regarding health care and policy implications, CQI has played a vital role in advancing PHC and fostering the realization of UHC goals worldwide. The indicators found in Donabedian’s framework that are positively influenced by CQI efforts are comparable to those included in the PHC performance initiative’s conceptual framework [29, 118, 119]. It is clearly explained that PHC serves as the roadmap to realizing the vision of UHC [120, 121]. Given these circumstances, implementing CQI can contribute to the achievement of PHC principles and the objectives of UHC. For instance, by implementing CQI methods, countries have enhanced the accessibility, affordability, and quality of PHC services, leading to better health outcomes for their populations. CQI has facilitated identifying and resolving healthcare gaps and inefficiencies, enabling countries to optimize resource allocation and deliver more effective and patient-centered care. However, it is crucial to recognize that the successful implementation of Continuous Quality Improvement (CQI) necessitates optimizing the duration of each cycle, understanding challenges and barriers that extend beyond the health system and settings, and acknowledging that its effectiveness may be compromised if these challenges are not adequately addressed.

Despite abundant literature, there are still gaps regarding the relationship between CQI and other dimensions within the healthcare system. No studies have examined the impact of CQI initiatives on catastrophic health expenditure, effective service coverage, patient-centredness, comprehensiveness, equity, health security, and responsiveness.

### Limitations

In conducting this review, it has some limitations to consider. Firstly, only articles published in English were included, which may introduce the exclusion of relevant non-English articles. Additionally, as this review follows a scoping methodology, the focus is on synthesising available evidence rather than critically evaluating or scoring the quality of the included articles.

## Conclusions

Continuous quality improvement is investigated as a continuous and ongoing intervention, where the implementation time can vary across different cycles. The CQI team and implementation timelines were critical elements of CQI in different models. Among the commonly used approaches, the PDSA or PDCA is frequently employed. In most CQI models, a wide range of tools, nineteen tools, are commonly utilized to support the improvement process. Cultural, technical, structural, and strategic barriers and facilitators are significant in implementing CQI initiatives. Implementing the CQI initiative aims to improve health system blocks, enhance health service delivery process and output, and ultimately prevent morbidity and reduce mortality. For future researchers, considering that CQI is context-dependent approach, conducting scale-up implementation research about catastrophic health expenditure, effective service coverage, patient-centredness, comprehensiveness, equity, health security, and responsiveness across various settings and health issues would be valuable.

### Supplementary Information

**Supplementary Material 1.**

**Supplementary Material 2.**

## Data Availability

The data used and/or analyzed during the current study are available in this manuscript and/or the supplementary file.

## References

[1] Shewhart WA, Deming WE (1967). Memoriam: Walter A. Shewhart, 1891–1967. Am Stat.

[2] Shewhart WA. Statistical method from the viewpoint of quality control. New York: Dover; 1986. ISBN 978-0486652320. OCLC 13822053. Reprint. Originally published: Washington, DC: Graduate School of the Department of Agriculture, 1939.

[3] Moen R, editor Foundation and History of the PDSA Cycle. Asian network for quality conference Tokyo. https://www.deming.org/sites/default/files/pdf/2015/PDSA_History_Ron_MoenPdf. 2009.

[4] Kuperman G, James B, Jacobsen J, Gardner RM (1991). Continuous quality improvement applied to medical care: experiences at LDS hospital. Med Decis Making.

[5] Singh J, Singh H. Continuous improvement philosophy–literature review and directions. Benchmarking: An International Journal. 2015;22(1):75–119.

[6] Goldstone J (1997). Presidential address: Sony, Porsche, and vascular surgery in the 21st century. J Vasc Surg.

[7] Radawski D (1999). Continuous quality improvement: origins, concepts, problems, and applications. J Physician Assistant Educ.

[8] Shortell SM, O’Brien JL, Carman JM, Foster RW, Hughes E, Boerstler H (1995). Assessing the impact of continuous quality improvement/total quality management: concept versus implementation. Health Serv Res.

[9] Lohr K. Quality of health care: an introduction to critical definitions, concepts, principles, and practicalities. Striving for quality in health care. 1991.

[10] Berwick DM (1992). The clinical process and the quality process. Qual Manage Healthc.

[11] Gift B (1992). On the road to TQM. Food Manage.

[12] Greiner A, Knebel E. The core competencies needed for health care professionals. health professions education: A bridge to quality. 2003:45–73. 25057657

[13] McCalman J, Bailie R, Bainbridge R, McPhail-Bell K, Percival N, Askew D et al. Continuous quality improvement and comprehensive primary health care: a systems framework to improve service quality and health outcomes. Front Public Health. 2018:6 (76):1–6. 10.3389/fpubh.2018.00076 PMC5874897 29623271

[14] Sheingold BH, Hahn JA (2014). The history of healthcare quality: the first 100 years 1860–1960. Int J Afr Nurs Sci.

[15] Donabedian A (1966). Evaluating the quality of medical care. Milbank Q.

[16] Institute of Medicine (US) Committee on Quality of Health Care in America. Crossing the Quality Chasm: A New Health System for the 21st Century. Washington (DC): National Academies Press (US). 2001. 2, Improving the 21st-century Health Care System. Available from: https://www.ncbi.nlm.nih.gov/books/NBK222265/. 25057539

[17] Rubinstein A, Barani M, Lopez AS (2018). Quality first for effective universal health coverage in low-income and middle-income countries. Lancet Global Health.

[18] Agency for Healthcare Reserach and Quality. Quality Improvement and monitoring at your fingertips USA,: Agency for Healthcare Reserach and Quality. 2022. Available from: https://qualityindicators.ahrq.gov/.

[19] Anderson CA, Cassidy B, Rivenburgh P (1991). Implementing continuous quality improvement (CQI) in hospitals: lessons learned from the International Quality Study. Qual Assur Health Care.

[20] Gardner K, Mazza D (2012). Quality in general practice - definitions and frameworks. Aust Fam Physician.

[21] Loper AC, Jensen TM, Farley AB, Morgan JD, Metz AJ (2022). A systematic review of approaches for continuous quality improvement capacity-building. J Public Health Manage Pract.

[22] Hill JE, Stephani A-M, Sapple P, Clegg AJ (2020). The effectiveness of continuous quality improvement for developing professional practice and improving health care outcomes: a systematic review. Implement Sci.

[23] Candas B, Jobin G, Dubé C, Tousignant M, Abdeljelil AB, Grenier S (2016). Barriers and facilitators to implementing continuous quality improvement programs in colonoscopy services: a mixed methods systematic review. Endoscopy Int Open.

[24] Peters MD, Marnie C, Colquhoun H, Garritty CM, Hempel S, Horsley T (2021). Scoping reviews: reinforcing and advancing the methodology and application. Syst Reviews.

[25] Arksey H, O’Malley L (2005). Scoping studies: towards a methodological framework. Int J Soc Res Methodol.

[26] Tricco AC, Lillie E, Zarin W, O’Brien KK, Colquhoun H, Levac D (2018). PRISMA extension for scoping reviews (PRISMA-ScR): checklist and explanation. Ann Intern Med.

[27] McGowan J, Straus S, Moher D, Langlois EV, O’Brien KK, Horsley T (2020). Reporting scoping reviews—PRISMA ScR extension. J Clin Epidemiol.

[28] Donabedian A. Explorations in quality assessment and monitoring: the definition of quality and approaches to its assessment. Health Administration Press, Ann Arbor. 1980;1.

[29] World Health Organization. Operational framework for primary health care: transforming vision into action. Geneva: World Health Organization and the United Nations Children’s Fund (UNICEF); 2020 [updated 14 December 2020; cited 2023 Nov Oct 17]. Available from: https://www.who.int/publications/i/item/9789240017832.

[30] The Joanna Briggs Institute. The Joanna Briggs Institute Reviewers’ Manual :2014 edition. Australia: The Joanna Briggs Institute. 2014:88–91.

[31] Rihal CS, Kamath CC, Holmes DR, Reller MK, Anderson SS, McMurtry EK (2006). Economic and clinical outcomes of a physician-led continuous quality improvement intervention in the delivery of percutaneous coronary intervention. Am J Manag Care.

[32] Ade-Oshifogun JB, Dufelmeier T (2012). Prevention and Management of Do not return notices: a quality improvement process for Supplemental staffing nursing agencies. Nurs Forum.

[33] Rubenstein L, Khodyakov D, Hempel S, Danz M, Salem-Schatz S, Foy R (2014). How can we recognize continuous quality improvement?. Int J Qual Health Care.

[34] O’Neill SM, Hempel S, Lim YW, Danz MS, Foy R, Suttorp MJ (2011). Identifying continuous quality improvement publications: what makes an improvement intervention ‘CQI’?. BMJ Qual Saf.

[35] Sibthorpe B, Gardner K, McAullay D (2016). Furthering the quality agenda in Aboriginal community controlled health services: understanding the relationship between accreditation, continuous quality improvement and national key performance indicator reporting. Aust J Prim Health.

[36] Bennett CL, Crane JM (2001). Quality improvement efforts in oncology: are we ready to begin?. Cancer Invest.

[37] VanValkenburgh DA (2001). Implementing continuous quality improvement at the facility level. Adv Ren Replace Ther.

[38] Loper AC, Jensen TM, Farley AB, Morgan JD, Metz AJ (2022). A systematic review of approaches for continuous quality improvement capacity-building. J Public Health Manage Practice.

[39] Ryan M (2004). Achieving and sustaining quality in healthcare. Front Health Serv Manag.

[40] Nicolucci A, Allotta G, Allegra G, Cordaro G, D’Agati F, Di Benedetto A (2008). Five-year impact of a continuous quality improvement effort implemented by a network of diabetes outpatient clinics. Diabetes Care.

[41] Wakefield BJ, Blegen MA, Uden-Holman T, Vaughn T, Chrischilles E, Wakefield DS (2001). Organizational culture, continuous quality improvement, and medication administration error reporting. Am J Med Qual.

[42] Sori DA, Debelew GT, Degefa LS, Asefa Z (2023). Continuous quality improvement strategy for increasing immediate postpartum long-acting reversible contraceptive use at Jimma University Medical Center, Jimma, Ethiopia. BMJ Open Qual.

[43] Roche B, Robin C, Deleaval PJ, Marti MC (1998). Continuous quality improvement in ambulatory surgery: the non-attending patient. Ambul Surg.

[44] O’Connor JB, Sondhi SS, Mullen KD, McCullough AJ (1999). A continuous quality improvement initiative reduces inappropriate prescribing of prophylactic antibiotics for endoscopic procedures. Am J Gastroenterol.

[45] Ursu A, Greenberg G, McKee M (2019). Continuous quality improvement methodology: a case study on multidisciplinary collaboration to improve chlamydia screening. Fam Med Community Health.

[46] Quick B, Nordstrom S, Johnson K (2006). Using continuous quality improvement to implement evidence-based medicine. Lippincotts Case Manag.

[47] Oyeledun B, Phillips A, Oronsaye F, Alo OD, Shaffer N, Osibo B (2017). The effect of a continuous quality improvement intervention on retention-in-care at 6 months postpartum in a PMTCT Program in Northern Nigeria: results of a cluster randomized controlled study. J Acquir Immune Defic Syndr.

[48] Nyengerai T, Phohole M, Iqaba N, Kinge CW, Gori E, Moyo K (2021). Quality of service and continuous quality improvement in voluntary medical male circumcision programme across four provinces in South Africa: longitudinal and cross-sectional programme data. PLoS ONE.

[49] Wang J, Zhang H, Liu J, Zhang K, Yi B, Liu Y (2014). Implementation of a continuous quality improvement program reduces the occurrence of peritonitis in PD. Ren Fail.

[50] Stikes R, Barbier D (2013). Applying the plan-do-study-act model to increase the use of kangaroo care. J Nurs Manag.

[51] Wagner AD, Mugo C, Bluemer-Miroite S, Mutiti PM, Wamalwa DC, Bukusi D (2017). Continuous quality improvement intervention for adolescent and young adult HIV testing services in Kenya improves HIV knowledge. AIDS.

[52] Le RD, Melanson SE, Santos KS, Paredes JD, Baum JM, Goonan EM (2014). Using lean principles to optimise inpatient phlebotomy services. J Clin Pathol.

[53] Manyazewal T, Mekonnen A, Demelew T, Mengestu S, Abdu Y, Mammo D (2018). Improving immunization capacity in Ethiopia through continuous quality improvement interventions: a prospective quasi-experimental study. Infect Dis Poverty.

[54] Kamiya Y, Ishijma H, Hagiwara A, Takahashi S, Ngonyani HAM, Samky E (2017). Evaluating the impact of continuous quality improvement methods at hospitals in Tanzania: a cluster-randomized trial. Int J Qual Health Care.

[55] Kibbe DC, Bentz E, McLaughlin CP (1993). Continuous quality improvement for continuity of care. J Fam Pract.

[56] Adrawa N, Ongiro S, Lotee K, Seret J, Adeke M, Izudi J. Use of a context-specific package to increase sputum smear monitoring among people with pulmonary tuberculosis in Uganda: a quality improvement study. BMJ Open Qual. 2023;12(3):1–6. 10.1136/bmjoq-2023-002314 PMC10414073 37558284

[57] Hunt P, Hunter SB, Levan D (2017). Continuous quality improvement in substance abuse treatment facilities: how much does it cost?. J Subst Abuse Treat.

[58] Azadeh A, Ameli M, Alisoltani N, Motevali Haghighi S (2016). A unique fuzzy multi-control approach for continuous quality improvement in a radio therapy department. Qual Quantity.

[59] Memiah P, Tlale J, Shimabale M, Nzyoka S, Komba P, Sebeza J (2021). Continuous quality improvement (CQI) institutionalization to reach 95:95:95 HIV targets: a multicountry experience from the Global South. BMC Health Serv Res.

[60] Yapa HM, De Neve JW, Chetty T, Herbst C, Post FA, Jiamsakul A (2020). The impact of continuous quality improvement on coverage of antenatal HIV care tests in rural South Africa: results of a stepped-wedge cluster-randomised controlled implementation trial. PLoS Med.

[61] Dadi TL, Abebo TA, Yeshitla A, Abera Y, Tadesse D, Tsegaye S (2023). Impact of quality improvement interventions on facility readiness, quality and uptake of maternal and child health services in developing regions of Ethiopia: a secondary analysis of programme data. BMJ Open Qual.

[62] Weinberg M, Fuentes JM, Ruiz AI, Lozano FW, Angel E, Gaitan H (2001). Reducing infections among women undergoing cesarean section in Colombia by means of continuous quality improvement methods. Arch Intern Med.

[63] Andreoni V, Bilak Y, Bukumira M, Halfer D, Lynch-Stapleton P, Perez C (1995). Project management: putting continuous quality improvement theory into practice. J Nurs Care Qual.

[64] Balfour ME, Zinn TE, Cason K, Fox J, Morales M, Berdeja C (2018). Provider-payer partnerships as an engine for continuous quality improvement. Psychiatric Serv.

[65] Agurto I, Sandoval J, De La Rosa M, Guardado ME (2006). Improving cervical cancer prevention in a developing country. Int J Qual Health Care.

[66] Anderson CI, Basson MD, Ali M, Davis AT, Osmer RL, McLeod MK (2018). Comprehensive multicenter graduate surgical education initiative incorporating entrustable professional activities, continuous quality improvement cycles, and a web-based platform to enhance teaching and learning. J Am Coll Surg.

[67] Benjamin S, Seaman M (1998). Applying continuous quality improvement and human performance technology to primary health care in Bahrain. Health Care Superv.

[68] Byabagambi J, Marks P, Megere H, Karamagi E, Byakika S, Opio A (2015). Improving the quality of voluntary medical male circumcision through use of the continuous quality improvement approach: a pilot in 30 PEPFAR-Supported sites in Uganda. PLoS ONE.

[69] Hogg S, Roe Y, Mills R (2017). Implementing evidence-based continuous quality improvement strategies in an urban Aboriginal Community Controlled Health Service in South East Queensland: a best practice implementation pilot. JBI Database Syst Rev Implement Rep.

[70] Hopper MB, Morgan S (2014). Continuous quality improvement initiative for pressure ulcer prevention. J Wound Ostomy Cont Nurs.

[71] Ji J, Jiang DD, Xu Z, Yang YQ, Qian KY, Zhang MX (2021). Continuous quality improvement of nutrition management during radiotherapy in patients with nasopharyngeal carcinoma. Nurs Open.

[72] Chen M, Deng JH, Zhou FD, Wang M, Wang HY (2006). Improving the management of anemia in hemodialysis patients by implementing the continuous quality improvement program. Blood Purif.

[73] Reeves S, Matney K, Crane V (1995). Continuous quality improvement as an ideal in hospital practice. Health Care Superv.

[74] Barton AJ, Danek G, Johns P, Coons M (1998). Improving patient outcomes through CQI: vascular access planning. J Nurs Care Qual.

[75] Buttigieg SC, Gauci D, Dey P (2016). Continuous quality improvement in a Maltese hospital using logical framework analysis. J Health Organ Manag.

[76] Take N, Byakika S, Tasei H, Yoshikawa T (2015). The effect of 5S-continuous quality improvement-total quality management approach on staff motivation, patients’ waiting time and patient satisfaction with services at hospitals in Uganda. J Public Health Afr.

[77] Jacobson GH, McCoin NS, Lescallette R, Russ S, Slovis CM (2009). Kaizen: a method of process improvement in the emergency department. Acad Emerg Med.

[78] Agarwal S, Gallo J, Parashar A, Agarwal K, Ellis S, Khot U (2015). Impact of lean six sigma process improvement methodology on cardiac catheterization laboratory efficiency. Catheter Cardiovasc Interv.

[79] Rahul G, Samanta AK, Varaprasad G A Lean Six Sigma approach to reduce overcrowding of patients and improving the discharge process in a super-specialty hospital. In 2020 International Conference on System, Computation, Automation and Networking (ICSCAN) 2020 July 3 (pp. 1-6). IEEE

[80] Patel J, Nattabi B, Long R, Durey A, Naoum S, Kruger E, et al. The 5 C model: A proposed continuous quality improvement framework for volunteer dental services in remote Australian Aboriginal communities. Community Dent Oral Epidemiol. 2023;51(6):1150–8. 10.1111/cdoe.12850 36812158

[81] Van Acker B, McIntosh G, Gudes M (1998). Continuous quality improvement techniques enhance HMO members’ immunization rates. J Healthc Qual.

[82] Horine PD, Pohjala ED, Luecke RW (1993). Healthcare financial managers and CQI. Healthc Financ Manage.

[83] Reynolds JL (1995). Reducing the frequency of episiotomies through a continuous quality improvement program. CMAJ.

[84] Bunik M, Galloway K, Maughlin M, Hyman D (2021). First five quality improvement program increases adherence and continuity with well-child care. Pediatr Qual Saf.

[85] Boyle TA, MacKinnon NJ, Mahaffey T, Duggan K, Dow N (2012). Challenges of standardized continuous quality improvement programs in community pharmacies: the case of SafetyNET-Rx. Res Social Adm Pharm.

[86] Price A, Schwartz R, Cohen J, Manson H, Scott F (2017). Assessing continuous quality improvement in public health: adapting lessons from healthcare. Healthc Policy.

[87] Gage AD, Gotsadze T, Seid E, Mutasa R, Friedman J (2022). The influence of continuous quality improvement on healthcare quality: a mixed-methods study from Zimbabwe. Soc Sci Med.

[88] Chan YC, Ho SJ (1997). Continuous quality improvement: a survey of American and Canadian healthcare executives. Hosp Health Serv Adm.

[89] Balas EA, Puryear J, Mitchell JA, Barter B (1994). How to structure clinical practice guidelines for continuous quality improvement?. J Med Syst.

[90] ElChamaa R, Seely AJE, Jeong D, Kitto S (2022). Barriers and facilitators to the implementation and adoption of a continuous quality improvement program in surgery: a case study. J Contin Educ Health Prof.

[91] Candas B, Jobin G, Dubé C, Tousignant M, Abdeljelil A, Grenier S (2016). Barriers and facilitators to implementing continuous quality improvement programs in colonoscopy services: a mixed methods systematic review. Endoscopy Int Open.

[92] Brandrud AS, Schreiner A, Hjortdahl P, Helljesen GS, Nyen B, Nelson EC (2011). Three success factors for continual improvement in healthcare: an analysis of the reports of improvement team members. BMJ Qual Saf.

[93] Lee S, Choi KS, Kang HY, Cho W, Chae YM (2002). Assessing the factors influencing continuous quality improvement implementation: experience in Korean hospitals. Int J Qual Health Care.

[94] Horwood C, Butler L, Barker P, Phakathi S, Haskins L, Grant M (2017). A continuous quality improvement intervention to improve the effectiveness of community health workers providing care to mothers and children: a cluster randomised controlled trial in South Africa. Hum Resour Health.

[95] Hyrkäs K, Lehti K (2003). Continuous quality improvement through team supervision supported by continuous self-monitoring of work and systematic patient feedback. J Nurs Manag.

[96] Akdemir N, Peterson LN, Campbell CM, Scheele F (2020). Evaluation of continuous quality improvement in accreditation for medical education. BMC Med Educ.

[97] Barzansky B, Hunt D, Moineau G, Ahn D, Lai CW, Humphrey H (2015). Continuous quality improvement in an accreditation system for undergraduate medical education: benefits and challenges. Med Teach.

[98] Gaylis F, Nasseri R, Salmasi A, Anderson C, Mohedin S, Prime R (2021). Implementing continuous quality improvement in an integrated community urology practice: lessons learned. Urology.

[99] Gaga S, Mqoqi N, Chimatira R, Moko S, Igumbor JO (2021). Continuous quality improvement in HIV and TB services at selected healthcare facilities in South Africa. South Afr J HIV Med.

[100] Wang F, Yao D (2021). Application effect of continuous quality improvement measures on patient satisfaction and quality of life in gynecological nursing. Am J Transl Res.

[101] Lee SB, Lee LL, Yeung RS, Chan J (2013). A continuous quality improvement project to reduce medication error in the emergency department. World J Emerg Med.

[102] Chiang AA, Lee KC, Lee JC, Wei CH (1996). Effectiveness of a continuous quality improvement program aiming to reduce unplanned extubation: a prospective study. Intensive Care Med.

[103] Chinnaiyan K, Al-Mallah M, Goraya T, Patel S, Kazerooni E, Poopat C (2011). Impact of a continuous quality improvement initiative on appropriate use of coronary CT angiography: results from a multicenter, statewide registry, the advanced cardiovascular imaging consortium (ACIC). J Cardiovasc Comput Tomogr.

[104] Gibson-Helm M, Rumbold A, Teede H, Ranasinha S, Bailie R, Boyle J (2015). A continuous quality improvement initiative: improving the provision of pregnancy care for Aboriginal and Torres Strait Islander women. BJOG: Int J Obstet Gynecol.

[105] Bennett IM, Coco A, Anderson J, Horst M, Gambler AS, Barr WB (2009). Improving maternal care with a continuous quality improvement strategy: a report from the interventions to minimize preterm and low birth weight infants through continuous improvement techniques (IMPLICIT) network. J Am Board Fam Med.

[106] Krall SP, Iv CLR, Donahue L (1995). Effect of continuous quality improvement methods on reducing triage to thrombolytic interval for Acute myocardial infarction. Acad Emerg Med.

[107] Swanson TK, Eilers GM (1994). Physician and staff acceptance of continuous quality improvement. Fam Med.

[108] Yu Y, Zhou Y, Wang H, Zhou T, Li Q, Li T (2014). Impact of continuous quality improvement initiatives on clinical outcomes in peritoneal dialysis. Perit Dial Int.

[109] Schiff GD, Goldfield NI (1994). Deming meets Braverman: toward a progressive analysis of the continuous quality improvement paradigm. Int J Health Serv.

[110] American Hospital Association Division of Quality Resources Chicago, IL: The role of hospital leadership in the continuous improvement of patient care quality. American Hospital Association. J Healthc Qual. 1992;14(5):8–14,22. 10120433

[111] Scriven M. The Logic and Methodology of checklists [dissertation]. Western Michigan University; 2000.

[112] Hales B, Terblanche M, Fowler R, Sibbald W (2008). Development of medical checklists for improved quality of patient care. Int J Qual Health Care.

[113] Vermeir P, Vandijck D, Degroote S, Peleman R, Verhaeghe R, Mortier E (2015). Communication in healthcare: a narrative review of the literature and practical recommendations. Int J Clin Pract.

[114] Eljiz K, Greenfield D, Hogden A, Taylor R, Siddiqui N, Agaliotis M (2020). Improving knowledge translation for increased engagement and impact in healthcare. BMJ open Qual.

[115] O’Brien JL, Shortell SM, Hughes EF, Foster RW, Carman JM, Boerstler H (1995). An integrative model for organization-wide quality improvement: lessons from the field. Qual Manage Healthc.

[116] Adily A, Girgis S, D’Este C, Matthews V, Ward JE (2020). Syphilis testing performance in Aboriginal primary health care: exploring impact of continuous quality improvement over time. Aust J Prim Health.

[117] Horwood C, Butler L, Barker P, Phakathi S, Haskins L, Grant M (2017). A continuous quality improvement intervention to improve the effectiveness of community health workers providing care to mothers and children: a cluster randomised controlled trial in South Africa. Hum Resour Health.

[118] Veillard J, Cowling K, Bitton A, Ratcliffe H, Kimball M, Barkley S (2017). Better measurement for performance improvement in low- and middle-income countries: the primary Health Care Performance Initiative (PHCPI) experience of conceptual framework development and indicator selection. Milbank Q.

[119] Barbazza E, Kringos D, Kruse I, Klazinga NS, Tello JE (2019). Creating performance intelligence for primary health care strengthening in Europe. BMC Health Serv Res.

[120] Assefa Y, Hill PS, Gilks CF, Admassu M, Tesfaye D, Van Damme W (2020). Primary health care contributions to universal health coverage. Ethiopia Bull World Health Organ.

[121] Van Weel C, Kidd MR (2018). Why strengthening primary health care is essential to achieving universal health coverage. CMAJ.

