# Supplementary material for: A scoping review of continuous quality improvement in healthcare system: conceptualization, models and tools, barriers and facilitators, and impact
Source: BMC Health Serv Res. 2024 Apr 19;24:487. doi: 10.1186/s12913-024-10828-0 (PMC11031995; doi:10.1186/s12913-024-10828-0)
Supplement: Supplementary file 1 — Supplementary Material 1. [file 12913_2024_10828_MOESM1_ESM.docx]

Supplementary file 1: Search strategy

PubMed

("Continuous Quality Improvement*"[Title/Abstract] OR "Continuous Quality Assurance*"[Title/Abstract] OR "Continuous Quality Management*"[Title/Abstract] OR "continuous process improvement*"[Title/Abstract] OR "continuous performance improvement*"[Title/Abstract]) AND (("health system*"[Title/Abstract] OR health[Title/Abstract] OR "health care*"[Title/Abstract] OR healthcare* [Title/Abstract] OR “primary healthcare*” [Title/Abstract] OR “primary health-care*” [Title/Abstract] OR “primary health care*” OR “Universal Health Coverage” [Title/Abstract] OR “universal coverage” [Title/Abstract] OR "Quality of Care"[Title/Abstract] OR "quality care"[Title/Abstract] OR Effect*[Title/Abstract] OR Efficienc*[Title/Abstract] OR equit*[Title/Abstract] OR cost[Title/Abstract] OR responsive*[Title/Abstract] OR access[Title/Abstract] OR "people centred care"[Title/Abstract] OR "people-centred care"[Title/Abstract] OR "people centered care"[Title/Abstract] OR coordination[Title/Abstract] OR collaboration[Title/Abstract] OR comprehensive*[Title/Abstract] OR "continuity of care"[Title/Abstract] OR satisfaction[Title/Abstract] OR mortality[Title/Abstract] OR recovery[Title/Abstract] OR outcome[Title/Abstract] OR impact[Title/Abstract] OR coverage[Title/Abstract] OR availabl*[Title/Abstract] OR safety[Title/Abstract] OR resilience[Title/Abstract] OR security[Title/Abstract])) = 2893

SCOPUS

 TITLE-ABS-KEY ( "Continuous quality improvement*"  OR "Continuous Quality Assurance*"  OR "Continuous Quality Management*"  OR "continuous process improvement*" OR "Continuous performance improvement*" ) AND TITLE-ABS-KEY( "health system*" ) OR TITLE-ABS-KEY ( health ) OR TITLE-ABS-KEY ( "health care*" ) OR TITLE-ABS-KEY ( healthcare* ) OR TITLE-ABS-KEY ( "primary healthcare*" ) OR TITLE-ABS-KEY ( "primary health-care*" ) OR TITLE-ABS-KEY ( "primary health care*" ) OR TITLE-ABS-KEY ( "Universal Health Coverage" ) OR TITLE-ABS-KEY ( "universal coverage) OR TITLE-ABS-KEY(" quality AND of AND care ") OR TITLE-ABS-KEY(" quality AND care ") OR TITLE-ABS-KEY(effect*) OR TITLE-ABS-KEY(efficienc*) OR TITLE-ABS-KEY(equit*) OR TITLE-ABS-KEY(cost*) OR TITLE-ABS-KEY(responsive*) OR TITLE-ABS-KEY(access*) OR TITLE-ABS-KEY(" people AND centred AND care ") OR TITLE-ABS-KEY(" people-centred AND care ") OR TITLE-ABS-KEY(" people AND centered AND care ") OR TITLE-ABS-KEY(coordination) OR TITLE-ABS-KEY(collaboration) OR TITLE-ABS-KEY(comprehensive*) OR TITLE-ABS-KEY(" continuity AND of AND care ) OR TITLE-ABS-KEY ( satisfaction ) OR TITLE-ABS-KEY ( mortality ) OR TITLE-ABS-KEY ( recovery ) OR TITLE-ABS-KEY ( outcome ) OR TITLE-ABS-KEY ( impact ) OR TITLE-ABS-KEY ( coverage ) OR TITLE-ABS-KEY ( available ) OR TITLE-ABS-KEY ( safety ) OR TITLE-ABS-KEY ( resilience ) OR TITLE-ABS-KEY ( security ) ) = 4339

Web of Science

#1: TI=("Continuous Quality Improvement*" or "Continuous Quality Assurance*" or "Continuous Quality Management*" or "Continuous process improvement*" or "Continuous performance improvement*) = 930

#2: TI=("health system*" or health or "health care*" or healthcare* or “primary healthcare*” or “primary health-care*” or “primary health care*” or “Universal Health Coverage” or “universal coverage” or "Quality of Care" or "quality care" or Effect* or Efficienc* or equit* or cost or responsive* or access or "people centred care" or "people-centred care" or "people centered care" or coordination or collaboration or comprehensive* or "Continuity of care" or satisfaction or mortality or recovery or outcome or impact or coverage or availabl* or safety or resilience or security ) = 9,258, 481

#3: #1 AND #2 = 225

EMBASE

#1: 'continuous quality improvement*':ab,ti OR 'continuous quality assurance*':ab,ti OR 'continuous quality management*':ab,ti OR 'continuous process improvement*':ab,ti OR 'continuous performance improvement*':ab,ti = 4476

#2: 'health system*' OR health OR 'health care*' OR healthcare* OR 'primary healthcare*' OR 'primary health-care*' OR 'primary health care*' OR 'universal health coverage' OR 'universal coverage' OR 'quality of care' OR 'quality care' OR effect* OR efficienc* OR equit* OR cost OR responsive* OR access OR 'people centred care' OR 'people-centred care' OR 'people centered care' OR coordination OR collaboration OR comprehensive* OR 'continuity of care' OR satisfaction OR mortality OR recovery OR outcome OR impact OR coverage OR availabl* OR safety OR resilience OR security = 18,283,250

#3: #1 AND #2 = 3,651
