# Supplementary material for: A scoping review of continuous quality improvement in healthcare system: conceptualization, models and tools, barriers and facilitators, and impact
Source: BMC Health Serv Res. 2024 Apr 19;24:487. doi: 10.1186/s12913-024-10828-0 (PMC11031995; doi:10.1186/s12913-024-10828-0)
Supplement: Supplementary file 2 — Supplementary Material 2. [file 12913_2024_10828_MOESM2_ESM.docx]

Supplementary file 2: Characteristics of included articles

| Author/year | Country | Health problem | purpose | Study design | Types of intervention | CQI models/approaches  (CQI tools) | Main findings |
| --- | --- | --- | --- | --- | --- | --- | --- |
| Ryan M/  2004 | USA | Not specified | implementation & strategies | Lead article | Not interventional: Lessons learned | Baldrige model (Tools not mentioned) | Strategies of CQI: apply Baldrige as a system, communication, research, feedback report, |
| Nicolucci A/2008 | Italy | Diabetes care in outpatient clinic | impact | Quantitative (interventional) | CQI efforts (e.g., electronic medical record systems) | Models not mentioned (Tables, pie-charts and histograms, boxplots, star-plots and variability graph) | Impact: increasing rates of monitoring, increasing drug prescription, and better levels of intermediate outcomes (glucose level control). |
| Ade-Oshifogun JB/2012 | None specified | Staffing in healthcare facilities | Application of CQI for staffing | Qualitative | Non interventional: Education, quiz, tool | Plan-Implement-Evaluate/Standardize-problem-cause (Tools not mentioned) | Activities were definition of areas for improvement, identification of all possible causes, development of an action plan, implementation of the action plan, evaluation for program improvement, and standardization of the process; improved staffing |
| Adily A/  2020 | Australia | Syphilis test for Aboriginal & Torres Strait Islander people | Impact of CQI on coverage | Quantitative (interventional) | A comprehensive CQI program (A preventive health audit module) | Audit-best practice for chronic disease (Systems assessment tools) | CQI cycles (two to nine times audit cycles) did not bring significant change to increase syphilis testing performance |
| Adrawa N/  2023 | Uganda | People with pulmonary tuberculosis in TB clinic | Impact of CQI on coverage | Quantitative (interventional) | Line listing of all eligible persons for sputum smear monitoring (SSM), use of reminder stickers to identify eligible persons for SSM, use of community health workers to conduct home visits for people with missed clinic visits, and integration of SSM into community-based ART points for distant persons | PDSA (Monthly data review and analysis) | The use of a context relevant CQI package was accompanied by improved sputum smear monitoring at 2, 5 and 6 months among people with bacteriologically confirmed pulmonary tuberculosis. |
| Agarwal S/  2015 | not specified | Elective & urgent cardiac catheterization in cardiac care | Impact of CQI service provision | Quantitative (longitudinal) | Value stream map drafted, preliminary data collection, inception of CQI team, recruitment of patient transporters and employee for Cath Lab cleaning in between cases, define responsibility for nurses and techs in the Cath Lab, training for staff and technicians to perform multiple tasks, Emphasis on utilization of the entire Cath Lab team for an efficient and expeditious turnover for the next case, Separate nursing personnel for the preparation–recovery area, to expedite patient arrival to the Cath Lab and patient exit from the Cath Lab after completion of the procedure, Ensuring a constant line of communication between the Cath Lab manager and the nursing supervisor of the preparation–recovery area to ensure a constant smooth flow of patients; Definition of performance metrics to monitor quality improvement , Establishment of goals based on these performance metrics; Implementation of daily huddle process — During the huddle, the Cath Lab manager would meet with all the nursing staff and techs for 15 min before the shift start and discuss the key performance metrics of the previous day. During this process, the group would try to identify the areas for potential improvement in efficiency and patient throughput; Implementation of a new sheath pull process and training of all nursing personnel in the recovery area for pulling sheaths; Development and implementation of monthly scorecards; Development and implementation of an interactive electronic patient tracking system with accessibility across the entire institution | Lean Six Sigma (A value stream map, Process flowchart) | Significant improvement in turn-time, physician downtime, on-time patient arrival, on-time physician arrival, on-time start as well as sheath-pulls inside the Cath Lab. |
| Agurto I/  2006 | developing countries | Cervical cancer in cancer clinic | impact of CQI on service delivery | Quantitative (interventional) | Involvement of policy, service provision and community levels in 4 plan-do-study-act cycles, facilitating linkages between work processes and a quality control group | Plan-do-study-act (Not mentioned) | Reduce turnaround time and increased follow-up |
| Akdemir N/  2020 | not specified | Medical education accreditation | Pros & cons of CQI in education institution | Qualitative | Non interventional: Seven criteria (serve the public interest, evaluate the benefits, examine the governance structure, enhance reflection, maintain impartiality and independence, be publicly accountable, balance expectations with capacity) | Both models and tools are not mentioned | CQI makes use of early warning systems, enhance the reflective function of accreditation, CQI has a potential role in safeguarding the public interest. However, CQI has weaknesses with respect to impartiality, independence, and public accountability, as well as with the ability to balance expectations with capacity. |
| Ursu A/  2019 |  | Chlamydia screening in healthcare facilities | impact on coverage | Quantitative (interventional) | Nine steps ((1) assemble a motivated team of stakeholders and leaders; (2) identify the problem that is considered a high priority; (3) prepare for the project including support and resources; (4) set a goal and ways to evaluate outcomes; (5) identify the root cause(s) of the problem and prioritise based on impact and effort to address; (6) develop a countermeasure that addresses the selected root cause effectively; (7) pilot a small-scale project to assess for possible modifications; (8) large-scale roll-out including education on how to implement the project; and (9) assess and modify the process with a feedback mechanism) | PDCA cycle (Tools not mentioned) | Using this nine-step process, chlamydia screening rates increased from 29% to 60%. |
| Anderson/  1991 | Canada, Germany, Japan, USA | Hospitals | concept | Qualitative (Lessons learned) | not mentioned | Both models and tools are not mentioned | I t was noted early on in the research that cultural variable dramatically shape the concept of quality. Two of the basic principles of Continuous Quality Improvement are creatin an organization-wide “quality definition " and a "patient orientation”. The quality definition needs to focus directly on the multiple consumers of the services provided. Therefore, in order to establish a quality definition, al l consumer s o f the services must be identified. I n the study we found that each culture addresses the definition from a different perspective. Americans focus the definition of quality around meeting patient and referring physician expectations. Th e German culture tends to focus mor e o n physician orientate d define d quality. Th e Canadian Health Car e System focused on universality, accessibility and comprehensibility. A second finding is the varying degree s of the physician' s control of the health car e delivery process. The preliminary results show direct correlations between cultural influences and the concept of quality. The perceived definition of quality by the various countries varies and therefore the application of "quality concepts" differs. There was identified ten principles (quality definition, patient orientation, supplier partnerships, work process focus, preventive systems, error-free attitudes, management by fact, employee empowerment, total organization, and continuous improvement) |
| Anderson C/  2018 | USA | Perioperative education in medical education | CQI on surgical education | Quantitative | Not mentioned | PDCA (Tools not mentioned) | Educational models with repeated Plan, Do, Check, Act cycles increased the quality of preoperative learning objectives, showed more frequent, detailed, and timely assessments of resident performance, and demonstrated more effective self-reflection by residents. |
| Andreoni V/  1995 | USA | General care in hospital |  | Case study | Seven steps (1) project start-up (clarify mission, develop workplan, identify customer needs), 2)current situation (describe current process, localize the problem, identify baseline data needed), 3) cause analysis (identify root cause), 4) solutions (generate/evaluate solutions to root causes, pilot/trial run), 5) results (evaluate pilot, compare with baseline data, study results, standardize or repeat cycle), 6) standardization (describe new process, methods for communicating provisions for training, monitoring, and evaluating), 7) future plans (continue or close project, what was learned). | PDCA (Brainstorming technique, multivoting, and Pareto chart) | Include member of project management team to a liaison member to the CQI steering committee facilitated integration of the PDCA framework, definitions used for team functions and roles into the hospital’s UQ plan; consistency in approach, structure, and language provided to build process improvement |
| Azadeh A/  2016 | Iran | not specific problem in radiotherapy department | Fuzzy method for CQI | Quantitative | Fuzzy multicontrol approach having 12 steps (problem definition, identifying potential error factors and mapping cause and effect chart, ranking error factors, identifying patient's health criteria given treatment process, defining a multiple criteria decision making problem for criteria ranking, determining solving method and criteria ranking, collecting the results of assessing most important criteria, presenting tabulated fuzzy data, determining the selected criteria's control limit, draw multicontrol chart, controlling the graph and data, review | PDCA (Fuzzy method, multicontrol chart) | Fuzzy method besides supervision creates necessary control for investigation of emergency matters. The fuzzy TOPSIS (Technique for Order Preference by Similarity to Ideal Solution), the decision has been taken. This decision can be considered as the best decision because with this approach all the expertise view have been intended. |
| Balas AE/  1994 | USA | not specified problem guidelines | Application | Quantitative | CQI program | Both model and tool are not mentioned | The difficulty of finding codes for conditions and procedures, the high rate of non-codable items, and the lack of recommended measures limit the applicability of published clinical practice guidelines to continuous quality improvement programs. |
| Balfour ME/  2018 | USA | Care and cost | Strategies for CQI | descriptive (not clear) | CQI to Structure Clinical Practice Guidelines | PDCA (Tools are not mentioned) | Provider-payer partnership resulted in better management of services, resulting in significant decreases in readmissions and in the number of high utilizers. |
| Barton A/  1998 | USA | Vascular access planning | improving patient outcome | Quantitative | Focus on vascular access planning, analyze data concerning intravenous (IV) therapy, develop a vascular access planning algorithm, and execute implementation of the algorithm | FADE (Fishbone diagram) | CQI cause less difficulty and less stress to start IV, reduce waiting time, and length of stay |
| Barzansky B/  2015 | USA, Canada, Republic of Korea, Taiwan | Medical education accreditation | Benefits and challenges of CQI in education | Qualitative | Non interventional: CQI on accreditation | Both model and tool are not mentioned | A functional CQI process should be focused directly on accreditation standards so as to result in the improvement of educational quality and outcomes, be feasible to implement, avoid duplication of effort and have both commitment and resource support from the sponsoring entity and the individual medical schools. |
| Benjamin S/  1998 | Bahrain | PHC | Application | Qualitative (project) | Non interventional: 6 steps (identify and define problems and/or opportunities, analyze for root cause, generate alternatives, select alternative and plan implementation, implement, and evaluate, standardize, and reinforce) | PDSA (Tools are not mentioned) | CQI maintain or reduce operation cost in PHC, supported with participation, empowerment, and accountability |
| Bennet CL/  2001 | not specified | Oncology care | Challenges | Qualitative | Not mentioned | PDCA (Tools are not mentioned) | Cultural (physician decline to be working member of CQI, technical (inadequate capitalization of project and subsequent insufficient support for CQI facilitators and data entry managers, immature electronic medical records or medical information systems), structural (Difficulties in physician-to-physician cooperation and synergies), and strategic (failure to recognize the effects of the program on patient care, Inability to select proper goals, create consensus, and encourage CQI; Failure to integrate CQI into organizational planning and practice goals; Inadequate financial or other positive reinforcement to physicians to enhance participation) obstacles to successful implementation of CQI |
| Bennett IM/  2009 | not specified | Maternal care | Impact of CQI | Quantitative (interventional) | Evidence review process involving independent working groups on selected problems, developed evidence-based interventions, Reviews were circulated and discussed via teleconference and collaboratively refined, prepare guideline, select prevention practice by consensus | Model is not mentioned (Charts, P-charts) | The CQI model improved maternal care (prenatal depression screening, screening for smoking at gestational period, contraception planning). |
| Boyle TA/  2014 | Canada | Perception on CQI outcome | Perception | Qualitative | Not mentioned | Both model and tool are not mentioned | Keeping the "continuous" in continuous quality improvement: exploring perceived outcomes of CQI program use in community pharmacy |
| Boyle TA/  2012 | Canada | Challenges of CQI | Challenges | Qualitative | Not mentioned | Both model and tool are not mentioned | Six major challenges were identified, specifically finding time to report, having all pharmacy staff involved in quality-related event (QRE) reporting, reporting apprehensiveness, changing staff relationships, meeting to discuss QREs, and accepting the online technology. Challenges were addressed in a number of ways including developing a manual-online hybrid reporting system, managers paying staff to meet after hours, and pharmacy managers showing visible commitment to QRE reporting and learning. |
| Brandrud AS/  2011 | Norway | Factors of CQI | Factors | Qualitative | Not mentioned | Both model and tool are not mentioned | Continuous and reliable information, including measurement, about test and current practice; engagement of everybody in all phases of the improvement work: the patient and family, the leadership, the professional environment and the staff; and an infrastructure based on improvement knowledge, with multidisciplinary teams, available coaching, learning systems and sustainability systems |
| Buttigieg SC/  2016 | United Kingdom | Hospital care | Application | qualitative | logical framework | Logical framework (Fishbone diagram, problem and objective tree) | identify systems/processes for improvement using brainstorming, develop problem tree through root cause analysis and casual relationship, develop objective tree through logical reasoning, formulate logical framework, plan, implement and operate improvement projects; logic framework improved quality in hospitals. |
| Byabagambi J/  2015 | Uganda | VMMC in care | impact of CQI on quality | Quantitative (interventional) | CQI program (Sites were supported to identify barriers in achieving national standards, identify possible solutions to overcome the barriers and carry out improvement plans to test these changes, while collecting performance data to objectively measure whether they had bridged gaps) | PDSA (Tools not mentioned) | At baseline (February-March 2013), less than 20 percent of sites scored in the “good” range (>80%) for supplies and equipment, patient counseling and surgical procedure; by November 2013, the proportion of sites scoring “good” rose to 67 percent, 93 percent and 90 percent, respectively. Significant improvement was noted in post-operative follow-up at 48 hours, sexually transmitted infection assessment, informed consent and use of local anesthesia but **not rate of adverse events** |
| Candas B/  2015 | not specified | Colonoscopy services | Barriers and facilitators to CQI | systematic review | CQI program | Both model and tool are not mentioned | The most reported facilitators to CQI implementation are perception of feasibility, adoption of a formative approach, training and education, confidentiality, and assessing a limited number of quality indicators. Receptive attitudes, a sense of ownership and perceptions of positive impacts also facilitate the implementation. Finally, an organizational environment conducive to quality improvement has to be inclusive of all user groups, explicitly supportive, and provide appropriate resources |
| Chan YC/  1997 | Canada and USA | Barriers and facilitators to CQI in healthcare facilities | Barriers and facilitators to CQI | Qualitative based on key informants | CQI program | Both model and tool are not mentioned | Lack of CQI skills, poor planning, and insufficient staffing were attributes of unsuccessful CQI efforts in Canadian and American healthcare |
| Chen M/  2006 | China | Anaemia care | Impact of CQI | Quantitative (interventional) | The CQI team find opportunities for improvement using data tracking, literature, and patient surveys; organize a dynamic multidisciplinary team composed of nephrologists and nurses; clarify the current process; create a flowchart of the current process, and assess these processes to determine whether they facilitate positive outcomes to the greatest extent possible; uncover and discuss weaknesses in the current process; start a plan of intervention | FOCUS-PDCA cycle (flowchart) | Implementation of CQI program is crucial to manage anaemia (i.e., the proportion of patients with an adequate blood iron and haemoglobin level significantly increased after CQI implementation) |
| Chiang AA /  1996 | USA | Incidence of unplanned endotracheal extubation for women | Effectiveness of CQI program | Quantitative (intervention) | CQI program focusing on standardization of procedures, improvement of communication, and identification and management of high-risk patients | Both model and tool are not mentioned | The implementation of a concerted CQI program is effective in reducing the overall incidence of unplanned endotracheal extubation |
| Chinnaiyan K/  2011 |  | Coronary computed tomography angiography care | Effect of CQI on use of imaging | quantitative (intervention) | CQI program (study initiation, site-specific plan, education plan, site-specific activities, data monitoring) | Both model and tool are not mentioned | CQI initiatives improve appropriate use of computed tomography angiography |
| ElChamaa R/  2022 | Canada | Surgery | Barriers and facilitators to CQI | Qualitative | CQI program (specific intervention is not mentioned) | Both model and tool are not mentioned | A key barrier was the lack of support from the hospital necessitating surgeons’ self-funding their own PMF programs. Facilitators included having a champion to drive CQI and using seminars to facilitate discussions around CQI principles and practices |
| Gaga S/  2021 | South Africa | HIV & TB care | Impact | Quantitative (interventional) | The interventions ranged from activities to improve drug procurement and dispensary procedures to clinical skills development, mentorship and supportive supervision. They also included improvements in monitoring and evaluation, and information utilisation for decision-making, targeted service improvement, service delivery campaigns and community engagement activities, support with patient flow management and human resource management support. | Both model and tool are not mentioned | The services identified as low quality were related to opportunistic infections management and laboratory practices. Compliance to prescribed service items in antiretroviral treatment initiation and monitoring, pharmacy and laboratory management, exceeded 70% across study sites. Over 80% of low-quality service delivery items were optimised in less than six months with targeted quality improvement support. |
| Gage AD/  2022 | Zimbabwe | ANC, PNC, PMTCT, Child assessment and treatment, maternal care, obstetric complications | Impact, barriers and facilitators | Mixed methods | Build capacity of facility health care teams, district managers, and supervisors; design the intervention; Define measurable improvement aims for priority best practices; Discuss, review, and prioritize changes to routine care processes to improve adherence with incentivized best practices; Collect and analyze monthly clinical quality measures using CQI checklist designed in the PBF project to assess progress against defined improvement aims; and Ensure regular posting of results in relevant patient care areas for public information | Model is not mentioned (Checklist) | Among the seven services assessed, CQI was associated with quality improvement in primary health centers for two: postnatal care and maternal delivery care. Enabling factors included strengthened leadership, teamwork and joint decision-making at facilities, and supportive supervision. Impeding factors included fragmentation of quality assurance policies; staff shortages and turnover; and gaps in the CQI training. Improvements were limited when considering the full breadth of potential outcomes but arise in certain areas of core focus of the CQI program. To see large scale improvement in the quality of healthcare in Zimbabwe, CQI should be seen as one potential tool in a broader health systems quality improvement strategy. |
| Gaylis F/  2020 | USA | Prostate cancer in Urology practice | impact of CQI | Quantitative (interventional) | CQI intervention (the impact of audited physician feedback on improving physicians’ adoption of active surveillance for low-risk prostate cancer (CaP) and adherence to a prostate biopsy time-out intervention) | Both model and tool are not mentioned | For active surveillance, we consistently saw an increase in active surveillance adoption for low-risk CaP patients in association with continuous audited feedback (P < .001). Adherence to the prostate biopsy time-out template improved when audited feedback was provided (P < .001). |
| Gibson-Helm ME/2016 | Australia | Pregnancy care for Aboriginal & Torres Strait Islander | Impact of CQI | Quantitative (interventional) | Delivery system design (e.g., continuity of care), information systems and decision support (e.g. evidence-based guidelines), self-management support (e.g. providing appropriate education and behavior change interventions), links with community and other services, and organizational influence and integration (e.g. organizational culture).) | Both model and tool are not mentioned | Participation in a CQI initiative by PHCs in Indigenous communities is associated with greater provision of pregnancy care regarding lifestyle-related risk factors (screening for cigarette, nutrition counseling, and folate prescription) |
| Hincapie AL/  2021 | USA | Community pharmacy | CQI regulation | Quantitative | CQI regulation characteristics (Monitor staff performance, equipment, facilities, and adherence to standards of practice, Manage known, alleged, and suspected medication errors that reach the patient, Report medication errors/QREs to the Board, Encourage population of national aggregate database, Regularly review the pharmacy’s aggregate data, Document quality improvements made, Complete a conduct audits/self-assessments relating to medication safety, Provide training to pharmacy staff on best practices in error/QRE management, Encourage open dialogue about errors and other activities | Both model and tool are not mentioned | CQI regulations by State Board of Pharmacies for community pharmacy practice in the USA includes Monitor staff performance, equipment, facilities, and adherence to standards of practice; Manage known, alleged, and suspected medication errors that reach the patient; Report medication errors/QREs to the Board; Encourage population of national aggregate database; Regularly review the pharmacy’s aggregate data; Document quality improvements made; Complete a conduct audits/self-assessments relating to medication safety; Provide training to pharmacy staff on best practices in error/QRE management; and Encourage open dialogue about errors and other activities, which all these elements were not equally implemented. |
| Hogg S/  2017 | Australia | Urban Aboriginal Community Controlled Health Service | Impact of CQI | Quantitative (Interventional) | CQI strategies (audit criteria): Staff demonstrate knowledge regarding the importance of CQI activity, identifying the potential enablers to CQI at their clinic; Staff have attended education and training sessions on CQI; Staff are actively involved with CQI activity; There is a multidisciplinary team approach to problem solving within the CQI process | PDSA (Tool is not reported) | The study found that the implementation of regular, formally organized CQI strategies does have an immediate impact on clinical practice, in this case, by increasing staff awareness regarding the uptake of influenza vaccination against regional targets. The Plan Do Study Act cycle is an efficient tool to record and monitor the change and to guide discussions. For the CQI process to be effective, continued education and training on data interpretation is pivotal to improve staff confidence to engage in regular data discussions, and this should be incorporated into all future CQI sessions. |
| Hopper MB/  2014 | USA | Pressure ulcer care | Impact of CQI | Quantitative (interventional) | CQI activities (evidence-based recommendations for process changes, initiate a basic treatment/prevention plan, mandatory hospital-wide education for all patient care staffs) | PDCA (Chart sticker) | 63% reduction in pressure ulcer prevalence from 2008 to 2010 |
| Horine PD/  1993 | Not specified | Not specified | Tools of CQI | Qualitative | Tools | Model is not mentioned (Brainstorming sessions, Check sheets, flowcharts, cause-and-effect diagrams, pareto diagram) | Describes CQI tools |
| Horwood C/  2017 | South Africa | Mothers & children care | Effectiveness of CQI program | Quantitative (RCT) | CQI-based mentoring | Both model and tool is not mentioned | At follow-up, compared to mothers served by control CHWs, mothers served by intervention CHWs were more likely to have received a CHW visit during pregnancy (75.7 vs 29.0%) and the postnatal period (72.6 vs 30.3%). Intervention mothers had higher maternal and child health knowledge scores (49 vs 43%) and reported higher exclusive breastfeeding rates to 6 weeks (76.7 vs 65.1%). HIV-positive mothers served by intervention CHWs were more likely to have disclosed their HIV status to the CHW (78.7 vs 50.0%). Uptake of facility-based interventions were not significantly different |
| Hunt P/  2017 |  | Substance abuse in substance abuse treatment facilities | impact of CQI on cost | Quantitative (interventional) | The timeline and activities of the CQI intervention were as follows: for the first three months, staff attended monthly CQI meetings to receive guidance on the “Plan” phase of PDSA. Next, key staff attended a fourth CQI meeting in which they identified and documented a “CQI Action” (i.e., a small improvement plan, consistent with the “Do” phase of PDSA). In the following monthly meetings, staff documented methods to study its impact (i.e., “Study” phases of PDSA), gave updates, and discussed any challenges with the PDSA approach or implementing their CQI Action. When the “Study” phase was completed, staff documented the “Act” phase, i.e., decided to continue, modify or revise the improvement plan | PSDA (Tool is not mentioned) | Our study finds implementation of CQI and meeting costs of this trial per facility were approximately $2000 to $10,500 per year ($4500 on average), or $10 to $60 per admitted client. While costs may be viewed as relatively low overall (less than 1% of total expenditures per year), some actions took more time and effort than others. |
| Hyrka¨s K/2003 | USA | Patient satisfaction & overall quality of care | impact of CQI | Quantitative (interventional) | The team supervision intervention | Model is not mentioned (control chart) | CQI integrated with team supervision improved patient satisfaction and the overall quality of care. |
| Jacobson GH/  2009 | USA | Emergency department | Application | Quantitative (interventional) | Kiazen principles ( Continually improve; No idea is too small; Identify, report, and solve individual problems; Focus change on common sense, low-cost, and low-risk improvements, not major innovations; Collect, verify, and analyze data to enact change; A major source of quality defects is problems in the process; Decreasing variability in the process is vital to improving quality; Identify and decrease non–value-added steps; Every interaction is between a customer and a supplier; Empower the worker to enact change; All ideas are addressed and responded to in some way; Decrease waste; Address the work place with good housekeeping discipline) | Kaizen (Tool is not mentioned) | There were 169, 105, and 101 KIs placed in the postimplementation calendar years 2006, 2007, and 2008, respectively. Seventy-six percent of KIs submitted thus far have identified a ‘‘process problem.’’ Fifty-three percent of KIs submitted have led to operational changes within the ED. Ninety-three percent of the resident physicians entered at least one KI, and 73% of these residents submitted more than one KI. Sixty-nine percent of the attendings entered at least one KI, and 89% of these attendings submitted more than one KI. |
| Ji J/  2021 | China | Nutritional management of patients with nasopharyngeal carcinoma in radiotherapy department | Impact of CQI | Quantitative | Find a process to improve (radiotherapy: the most common adverse reaction to radiotherapy is oral mucositis, which leads  to a decrease in food intake); organize a team that knows the process (Set up a quality improvement team that includes doctors, nurses with expertise in nutrition, bedside nurses, dieticians and pharmacists); clarify current knowledge of the process and identify existing processes (nutritional support was provided to educate patients about disease); understand the cause(s) of any variation in process (summarise the causes of malnutrition during radiotherapy); select the process improvement through a comprehensive literature search. Then, Plan the improvement and the optimal approach to continued efforts at data collection; Do the improvement, data collection and analysis; Develop normative nutrition support programs in five categories as follows: Patients diagnosed with NPC undergo nutritional screening, assessment and evaluation. C: Check and study the results. The head nurse regularly checks the nurses' implementation of the new procedures; Act to maintain improvements in the quality of care administered and to further improve the process. | FOCUS-PDCA (Tool is not mentioned) | The study found that the CQI model can decrease the severity of oral mucositis caused by radiation and improve nutritional status in affected patients. |
| Kamiya Y/  2016 | Tanzania | Hospitals outpatient departments | impact of CQI | Quantitative (RCT) | Implementation of the 5S (Sort, Set, Shine, Standardize, Sustain) over 12 months | 5S (Tool is not mentioned) | The 5S increased cleanliness in the outpatient department, patients’ subjective waiting time and overall satisfaction. However, negligible effects were confirmed for patient’s experiences on hospital staff behaviours. |
| Kibbe DC/  1993 | USA | Family practice centre | Impact of CQI on continuity of care | Quantitative (Interventional | Find a quality improvement opportunity; Organize a team that knows the processes involved; Clarify current knowledge of die process and its variation; Understand causes of process variation. Select the process improvement; Plan the process improvements. Do the data collection, analysis, and improvements; Study die data for process improvement and customer outcome; Act to hold the gains made. | FOCUS-PDSA (tool is not mentioned) | Process improvements were selected in the critical pathways that influence provider continuity. One year after implementation of selected process improvements, repeat chart audit showed that provider continuity levels had improved from 0.45 to 0.74, a 64% increase from 1 year earlier |
| Krall SP/  1995 | not specified | Acute myocardial infarction Triage | Impact of CQI on triage | Quantitative (interventional) | Interventions included: a triage protocol, CQI review, and staff feedback | Both model and tool are not mentioned | The implementation of CQI techniques, including 100% chart review, intensive systems analysis, and staff feedback, had a positive effect on the timeliness of thrombolytic therapy for the ED  patients who had AMI. As a result, most (79%) of the patients received therapy within the 60-minute time window recommended currently by the American Heart Association. |
| Le RD/  2014 | USA | Phlebotomy service | Optimize service | Quantitative (interventional) | Four Lean process improvement events and implemented a number of interventions in inpatient phlebotomy over a 9-month period | Lean (Tool is not mentioned) | Continuous process improvement through Lean can optimise workflow, improve timeliness, and decrease error in inpatient phlebotomy |
| Lee S/  2002 | Korea | Hospitals | Factors influencing CQI implementation | Quantitative | The Malcolm Baldrige National Quality Award Criteria (MBNQAC | Baldrige criteria (tool is not mentioned) | The average CQI implementation score across the seven dimensions by MBNQAC was 3.34 on a 5-point scale. The highest score was achieved in the dimension of ‘customer satisfaction’ (3.88), followed by ‘information/analysis’ (3.59), and ‘quality management’ (3.35). Regression analysis showed that hospitals which better fulfilled technical requirements, such as improving information systems, using more scientific CQI tools, and adopting systematic problem-solving approaches, tended to achieve higher degrees of CQI implementation. |
| Lee SB/  2013 | China | Emergency department | impact of CQI on medication error | Quantitative (intervention) | Phase 1 (problem identification): error-prone areas and intricate medication item lists. Phase 2 (problem elimination): Assign members of the task group to review the error-prone areas, streamline different medication item lists to eradicate duplicated and seldom used items, Elimination of medications that may be  inadvertently administered, and Standardization of practice. Phase 3 (assure sustainability): To conduct regular audit and to establish checking and review system. | Both model and tool are not mentioned | Responsible officers were assigned to look after seven error-prone areas. Strategies were proposed, discussed, endorsed and promulgated to eliminate the problems identified. A reduction of medication incidents (MI) from 16 to 6 was achieved before and after the improvement work. |
| Yapa HM/  2020 | South Africa | Antenatal HIV care | Impact of CQI | Quantitative (RCT) | CQI mentor and tools | PDSA cycles and action learning sessions (process maps, fishbone diagrams, run charts) | In Model 1, CQI significantly increased viral load monitoring but did not improve repeat HIV testing. |
| Manyazewal T/  2018 | Ethiopia | Immunization capacity in healthcare facilities | Impact of CQI | Quantitative (Quasi experimental) | Procedures included baseline quality assessment of immunization programme and services using structured checklists; immunization systems strengthening using onsite technical support, training, and supportive supervision interventions in a PDCA cycle over 12 months; and collection and analysis of data at baseline and at the 6th and 12th month of interventions | PDCA (checklists) | Following the interventions, vaccination coverage improved significantly from 63.6% at baseline to 79.3% for pentavalent, 62.5 to 72.8% for measles, 62.4 to 73.5% for BCG, 65.3 to 81.0% for PCV, and insignificantly from 56.2 to 74.2% for full vaccination. |
| Memiah P/  2021 | Kenya, Tanzania, Botswana, Zambia, Nigeria and Rwanda | 95-95-95 HIV care | Impact of CQI | Quantitative (interventional) | Analysing the problem to identify goals and objectives for improvement; developing individual changes or ‘change packages’; developing a monitoring system to measure improvements; and implementing and measuring changes | PDSA (Tool is not mentioned) | Adopting CQI processes is a useful approach for accelerating progress towards the attainment of the global 95:95:95 HIV targets; an increased partner testing acceptance rate from 21.7 to 48.2 % in Rwanda, which resulted in an increase in the HIV testing yield from 2.1 to 6.3 %. In Botswana, the overall linkage to treatment improved from 63 to 94 %, while in Kenya, the viral load testing uptake among paediatric and adolescent patients improved from 65 to 96 %, and the viral load suppression improved from 53 to 88 %. |
| Nyengerai T/  2021 | South Africa | VMMC care | Impact of CQI action plans | Quantitative (interventional) | Form CQI support teams (made-up of a clinical associate mentor, professional nurse mentor, enrolled nurse mentor, counsellor mentor and a data quality mentor); Baseline assessments were done; CQI support was provided; Routine data feedback meetings were conducted monthly to review progress against quality improvement plans developed. In addition, site improvement monitoring systems (SIMS); CQI focal persons were identified at each VMMC site, and these were assigned with responsibilities to focus on quality improvement requirements. Stakeholders for the VMMC program were also invited to share their success stories and barriers to program implementation as part of CQI support intervention. Following CQI implementation, post-test re-assessments were performed | PDSA cycle (time series charts, run charts and indicator monitoring) | Results showed significant increases for the overall changes in quality of service after CQI support intervention of 12% for infection prevention; 8% for male circumcision surgical procedure; 14% for individual counselling and HIV testing; 8% for group counselling, registration and communication; and 35% for monitoring and evaluation. In addition, there were significant increases for management systems of 29%, leadership and planning 23%, and supplies, equipment, environment and emergency 5%,. The overall quality of service performance across provinces increased by 18%. |
| O’Connor JB/  1999 | USA | Medication error | Impact of CQI initiatives for inappropriate prescription | Quantitative | Attendance at monthly divisional CQI meetings is mandatory for all staff gastroenterologists, gastroenterology fellows, and endoscopy nursing staff at our institutions. The committee reviews all major endoscopy related complications for the previous month. Quality issues relating to any aspect of the Gastroenterology Division are discussed. Using these existing monthly CQI meetings as a mechanism to initiate change in physician prophylactic antibiotic prescribing patterns, a senior staff member reviewed the literature on endoscopy associated bacteremic complications, the efficacy of antibiotics in preventing these infections, and the guidelines of United States and European societies on antibiotic prophylaxis | Both model and tool are not mentioned | The adoption of a divisional CQI initiative significantly reduced the administration of inappropriate prophylactic antibiotics. |
| O’Neill SM/  2011 | not specified | not specified | CQI features | Qualitative expert panel | CQI features assessment | Both model and tool are not mentioned | Feedback of systematically collected data’ was the most common feature (64%), followed by being at least ‘somewhat’ adapted to local conditions (61%), feedback at meetings involving participant leaders (46%), using an iterative development process (40%), being at least ‘somewhat’ data driven (34%), and using a recognised change method (28%). All six features were present in 14.2% of QII articles. |
| Oyeledun B/  2017 | Nigeria | PMTCT program | Impact of CQI on PMTCT care retention | Quantitative (RCT) | Establish CQI teams (made up of facility head/medical officer, study nurses/community health extension workers, and laboratory technicians), identify priority areas for improvement related to the 3 main drivers and then used the Plan-Do Study-Act model to formulate action plans, agree on indicators, collate and analyze facility data, and thereby test ideas for improving these specific areas. CQI coaches visited every 2 weeks to guide implementation of change ideas, including plot run-charts (line graphs used to identify and display trends over time) and provide structured assessment and response tools; technical assistance was also provided by phone. | PDSA (run charts) | There was no significant difference in retention at 6 months between the intervention and control arms [44% vs. 41%, relative risk: 1.08; 95% confidence interval (CI): 0.78 to 1.49]. Initiation of ARV prophylaxis among infants within 72 hours was not different by study arm (66.0% vs. 74.7%, relative risk = 0.95; 95% CI: 0.84 to 1.07) but rates of early infant testing at 4–6 weeks were higher in intervention sites (48.8% vs. 25.3%, adjusted relative risk: 1.76; 95% CI: 1.27 to 2.42). |
| Patel J/  2022 | Australia | Volunteer dental services | CQI framework | Qualitative | A cyclic 5C- model (consultation and moving through the phases of data collection, consideration, collaboration and celebration) | PDSA and 5C-model (not mentioned) | Interrogating the literature revealed five key themes: consultation, collection, consideration, collaboration and celebration all deemed to be integral to CQI for volunteer dental services to remote Aboriginal communities. Evidence synthesis then led to the proposed cyclical 5C framework, |
| Price A/  2017 | Canada | Conceptualize | Four dimensions of CQI | Qualitative | Four dimensions of CQI: strategic, Cultural, Technical, structural | Both model and tool are not mentioned | Strategic facilitators: leadership & communication, include all stakeholders in decision-making; Strategic barriers: time constraints, goals and priorities of leadership and management diverge, resource inadequacy, work overload; Cultural dimension facilitators: foster openness, collaboration, teamwork, and learning, developmental culture, group culture; cultural dimension barriers: resistance to change, achievements not celebrated or rewarded, hierarchical culture, rational culture; technical dimension facilitators: training opportunities, data quality and availability; technical dimension barriers: lack of training opportunities, poor information systems; structural dimension facilitator: effective forums of communication; structural dimension barriers: lack of mechanisms for disseminating knowledge, limited use of communication mechanisms |
| Quick B/  2006 | USA | Conceptualize | CQI for evidence-based practice | Qualitative | Not specified | Both model and tool are not mentioned | Seven-step process for implementing evidence-based practice using CQI include identifying the opportunity, assessing the current situation, analysis, proposed solutions, results, standardization, and future plans |
| Rahul G/  2020 | India | Emergency department Super-speciality hospital | Reduce overcrowding & improve discharge processes | Quantitative (interventional) | Lean Six Sigma approach | Lean Six Sigma, DMAIC (Why-why analysis, cause-and-effect diagram) | The simulation results show a significant reduction in the time gap between discharge orders and patient physically leaving emergency department from 76 minutes to 22 minutes. Hence, discharge process waiting time was reduced by 71%. |
| Reeves S/  1995 | USA | Hospital practice | Application | Quantitative (intervention) | FOCUS-PDCA approach was applied | FOCUS PDCA (Tool is not mentioned) | Reduce service for time and increase average daily volume of prescription by 32%; $160,000 per 6 months reduced wastage (reduced) |
| Reynolds JL/  1995 | Canada | Frequency of episiotomy in hospital | Impact of CQI | Quantitative | Identifying the opportunity for improvement (reduction in episiotomy rates), key "customers" in the process were identified as pregnant women, family physicians, nursing staff and consultant obstetricians. Initially the team spent much of its time mapping the current process of care, lined in CQI methodology". The key outcome measures and data to be collected were determined to be rates of episiotomies, perineal tears and extensions. | Model is not mentioned (Tool is flow chart) | The overall episiotomy rate decreased significantly from 44.5% to 33.3%. Among the primiparous women, the rate decreased from 57.6% to 46.2% and among the multiparous women from 34.3% to 23.6%. |
| Rihal CS/  2006 | not specified | Percutaneous Coronary Intervention | Impact of CQI on cost and clinical outcome | Quantitative (interventional) | The PDSA model begins with improvement teams asking themselves 3 important questions: (1) What are we trying to accomplish? (2) How will we know that a change is an improvement? (3) What changes can we make? | PDSA (Tool is not mentioned) | Compared with patients treated pre-CQI, those treated post-CQI had a reduced adjusted odds ratio for in-hospital death or any myocardial infarction (odds ratio = 0.66; 95% confidence interval = 0.46, 0.95). Models predicted a mean postprocedural length of stay difference of 0.8 days (2.8 days pre-CQI vs 2.0 days post-CQI; <.001) and an average post-CQI cost savings of $5430 (<.001) |
| Roche B/  1998 | not specified | Ambulatory surgery | Impact of CQI on decreasing non-attending patient | Quantitative (interventional) | A CQI programme is based on five points: 1. productive work is made through processes; 2. quality defects result in process problems; 3. quality improvement requires total employee involvement; 4. quality is customer focused; 5. CQI is undertaken by means of scientific and statistical analysis. Steps of CQI consists of analysing the actual situation: identify a process to improve; organise a team (physicians, anaesthesiologists, surgeons, nurses, hospital employees and managers) to work on the problem; clarify current knowledge of the process, identify data to be collected and collect data, including the potential contribution of the variable causes of the problem; understand the causes of process variation in analysing and delineating the potential causes of the problem; select the process improvement. Then, plan improvement after analysis of the most influential causes needing improvement; do improvement carrying out quality action; check data for process improvement; act to hold gain and continue improvement. To obtain an effective CQI process, it is mandatory to continue periodic monitoring. | FOCUS-PDCA (Tool is not mentioned) | The initial phase of the CQI study (FOCUS) ran for a period of 6 months. During this time, 967 patients underwent day surgery in our unit. Of these patients, 38 (3.9%) did not attend on the day of surgery. In the second phase (PDCA), the number of non-attending patients decreased in 1995 to 1.1% and in 1996 to 0.9% |
| Rubenstein L/  2014 | not specified | Conceptualize | Conceptualize | Qualitative | Not specified | Both model and tool are not specified | Features of CQI methods are systematic data guided activities, aiming to change routine work processes, creating a culture of quality improvement, specific predefined aims, using evidence relevant to the problem, designing with local conditions in mind, iterative development and testing, multidisciplinary teams from target organizations, data feedback to implementers, specific named improvement methods, and set of specific changes |
| Sibthorpe B/  2016 | Australia | Aboriginal community-controlled health services | Conceptualize | Qualitative | Not specified | PDSA (Tool is not mentioned) | CQI: focuses on improving client care and outcomes, determine by local needs and priorities, internally assessed, prospective and ongoing review, data for dialogue; measures, including performance indicators, with changeable targets, results vary over time, short cycles; quality internally assessed; data for dialogue and action; data published internally, shared among networks; wide range of measures that may include performance indicators (+/ informal, changeable targets and benchmarks); addresses any health issue |
| Sori DA/  2023 | Ethiopia | Postpartum long-acting reversible contraceptive (LARCs) use | Impact of CQI strategy | Quantitative (interventional) | Baseline data analysis, establish quality improvement team, CQI team prioritized the problems identified from baseline assessment, generated change ideas and  implemented the change ideas to see the progress of immediate postpartum LARCs’ uptake using monitoring, auditing and feedback tools over the next 8 weeks. Then, training of healthcare professionals (midwives and residents) on total quality of care concepts and family planning with a special focus on post-partum, and availing LARCs at each service delivery point 24/7 was done. | PDSA (Tool is not mentioned | Overall, through CQI intervention, the uptake of LARCs use was increased from 6.9% at the baseline to 25.37% over another 8 weeks of the intervention period. |
| Stikes R/  2013 |  | Kangaroo mother care | Impact of CQI | Quantitative (intervention) | Plan (identification problem, defined process, reviewed classic/current literature, developed surveys and kangaroo care education workshop, developed kangaroo care guidelines, designed parent education materials, obtained organizational support), Do (conducted pre-workshop survey and compiled results, Hosted kangaroo care education and workshop, Study (analysed pre-workshop survey results, monitored staff participation in kangaroo care, monitoring feedback on kangaroo care guidelines, equipment, and patient participation; conducted post-workshop survey and analyzed results), Act ( provided ongoing education, revised kangaroo care guidelines, continued audits of nursing practice, implemented kangaroo care program on labor and deliver and mother-baby units) | PDSA (Tool is not mentioned) | Four months post-implementation, the use of kangaroo care increased by 31%. |
| Take N/  2015 | Uganda | Staff motivation, patients' waiting time and patient satisfaction | impact of 5SCQI | Quantitative (interventional) | 5S related activities | 5S-CQI (Tool is not mentioned) | The study for Regional Referral Hospitals revealed 5S practice had the effect on staff motivation in terms of commitment to work in the current hospital and waiting time in the dispensary in 10 hospitals implementing 5S, but significant difference was not identified on patient satisfaction. The study for General Hospitals indicated the effect of 5S practice on patient satisfaction as well as waiting time, but staff motivation in two hospitals did not improve. 5S practice enables the hospitals to improve the quality of services in terms of staff motivation, waiting time and patient satisfaction and it takes as least four years in Uganda. |
| Van Acker B/  1998 | not specified | Immunization capacity in hospitals | Effect of Immunization rates | Quantitative (interventional) | Meeting: Review meeting objectives, Confirm and assign meeting roles, provide an overview of the agenda, Complete the work items; identify which meeting outputs are to be retained; Plan the next action steps and set the agenda for the next; Evaluate the meeting | Meeting (Flowcharts, affinity diagrams, multivoting, cause and effect/fish bone diagrams, and brainstorming  sessions) | The percentage of 1-year-olds who were fully immunized for DTP, OPV, and HIB is 89% in 1996, compared with 72.6% in 1995 and 73.6% in 1994 |
| VanValkenburgh DA/2001 | not specified | implementation | conceptualizing | qualitative | Not specified | PDCA (Tool is not mentioned) | The four building blocks of CQI program are: leadership, planning, quality control, and quality improvement. Leadership activities involves committing to the philosophy, establishing a mission statement, empowering the employees; planning activities are identifying internal and external customers and their needs, selecting CQI methodology, providing training to the staff, identifying outcome performance measures, adopting a formal CQI plan; quality control focus are establishing quality assurance parameters, collecting data, conducting CQI meeting, compare data to the performance measures; quality improvement include convene a quality improvement team and implement a CQI model. |
| Wagner AD/  2017 | Korea | HIV testing, HIV knowledge, satisfaction, intent to retest in clinics | impact of CQI | Quantitative (interventional) | Interventions are CQI training, supervision, and support | PDCA ( run charts) | CQI was associated with an immediate increase in the proportion of adolescent and young adults with accurate knowledge of HIV transmission at Youth Centre: 18 vs. 63%, and a trend at VCT: 38 vs. 72%. CQI was associated with an increase in the proportion of AYA with accurate HIV prevention knowledge in VCT: 46 vs. 61%, but not Youth Centre. In VCT, CQI showed a trend towards increased intent to retest (4.0 vs. 4.3), but not at Youth Centre. CQI was not associated with changes in AYA satisfaction, which was high during baseline and intervention at both clinics. |
| Wakefield BJ/  2001 | not specified | Medication error | Barriers and facilitators | Quantitative | Not specified | Both model and tool are not mentioned | A group-oriented culture positively correlated, while hierarchical and rational culture were negatively correlated with CQI implementation |
| Wang F/  2021 | not specified | maternal care in gynaecological nursing | effect on patient satisfaction and quality of life | Quantitative | The specific care was as follows: (1) A special CQI nursing team was established and led by a quality controller and head nurses. Besides, relevant nursing staff served as members of the team and were trained specially in the preparatory work. (2) During the process of actual implementation, it was necessary to comprehensively summarize the existing problems, mainly including the common errors of nursing staff in their work. (3) Potential risks were evaluated. (4) The principle of three inspections and seven verifications was strictly implemented. (5) Ward management was strengthened. (6) The work content of nursing staff was complicated. In this case, their psychology was prone to change, which also required full attention. | Both model and tool are not mentioned | CQI measures can optimize care quality management, improve the quality of clinical gynecological nursing, reduce nursing defects and improve both the maternal and newborn status, thus increasing patient satisfaction and improving quality of life |
| Wang J/  2014 | China | Peritonitis incidence in hospitals | Impact of CQI on peritonitis incidence | Quantitative (interventional) | Establishing a CQI team, training to CQI team, identification of risk factor, and action plan | PDCA (Tool is not mentioned) | The overall incidence of peritonitis reduced from once every 40.1 patient months before the CQI to once every 70.8 patient months after CQI. The incidence of Grampositive bacteria peritonitis reduced from once every 96.9 patients per month before CQI to once every 209.1 patient months after CQI, whereas the incidence of Gram-negative bacteria peritonitis reduced from once every 234.2 patient months before CQI to once every 292.8 patient months after CQI. |
| Weinberg M/  2001 | Colombia | Caesarean Section in hospitals | Impact of CQI to prevent infection | Quantitative (interventional) | CQI methods: three common questions, team training, brainstorming. | PDSA (Cause-and-effect diagram/fishbone or Ishikawa diagram) | CQI increased the overall and timely administration of prophylaxis; prophylaxis coverage increased from 36% to 89%, and timely administration increased from 50% to 96%; the infection rate was significantly declined. |
| Wendwessen N/  2020 | Ethiopia | Implementation in healthcare facilities | Determinants | Quantitative (cross-sectional) | not specified | Both model and tool are not mentioned | “Leaders receptiveness to new ideas”, “Leaders share information/data about health facility service delivery status”, “Health facility has a quality improvement project plan”, “Staff know using indicators to tell progress about service delivery”, and “Health facility assess client satisfaction level” were found to be independent predictors of continuous quality improvement (CQI) project implementation |
| Yu Y/  2013 | China | Peritoneal dialysis in health facilities | Impact of CQI on peritoneal dialysis | Quantitative (retrospective interventional) | not specified | Both model and tool are not mentioned | After implementation of the CQI initiative, the peritonitis rate declined to 1 episode in 77.25 patient–months from 1 episode in 22.86 patient–months. Ultrasound parameters of cardiac structure were generally unchanged in the CQI group, but significant increases in cardiothoracic ratio and interventricular septal thickness were observed in the control group (both p < 0.05). Patient survival at 1, 2, and 3 years was significantly higher in the CQI group (97.3%, 96.3%, and 96.3% respectively) than in the control group (92.6%, 82.4%, and 67.3% respectively, p < 0.001). Implementation of the CQI initiative also appeared to significantly improve technique survival rates: 95.6%, 92.6%, and 92.6% in the CQI group compared with 89.6%, 79.2%, and 76.8% in the control group (p < 0.001) after 1, 2, and 3 years respectively. |
| Bunik M/  2021 | USA | Post-natal follow-up in healthcare facilities | Impact of CQI on continuity of care | Quantitative (interventional) | Interventions targeted modification of electronic medical record templates, scheduling, staff and parental education, standardization of work processes, and birth to 1-year age-specific incentives | PDSA (run charts) | At baseline in 2012, completion of all 5 visits was in only 25% of the children; <10% of those children had consecutive visits with the same provider. After multiple Plan-Do-Study-Act cycles and pilot, the "First Five" well-childcare adherence rose to 78%, and continuity increased to 74% in 2018 |
| Clark DM/2013 | United Kingdom | Laboratory medicine | Principles of CQI | Leading article | Describing the application of Lean management systems to Laboratory Medicine | Lean (Tools are not mentioned) | CQI systems based on Lean principles combine systematic process improvement to improve the quality of service for users and patients and a commitment to value and develop the skills of the staff who deliver the service. CQI cultures require leadership that is committed to practicing and coaching the Plan-Do-Check-Act cycle of improvement and embedding it in their management systems. |
| Radawski D/1999 | Not specified | Physician assistant program | Origins, concepts, problems, and application | Special article | Not interventional: describes origins, concepts, problems, and applications | PDCA (Tool is not mentioned) | CQI is a style of management philosophy and method. In an adult world of stress in the competing spheres of life it offers both leaders and employees a positive, professional, kind, and respectful way to communicate and interact in the workplace. It also objectively evaluates and continuously improves the ways in which a business is structured and developed. Continuous quality improvement may have applications in physician assistant and other professional, personal, and family environments. |
| Shortell SM/1995 | USA | Hospitals | Concept and implementation | Qualitatively addressed | Examines the relationships among organizational culture, quality improvement processes and selected outcomes | Baldrige criteria (cause-and-effect diagrams, pareto charts) | A participative, flexible, risk-taking organizational culture was significantly related to quality improvement implementation. Quality improvement implementation was positively associated with greater perceived patient outcomes and human resource development. Larger-size hospitals experienced lower clinical efficiency with regard to higher charges and higher length of stay, due in part to having more bureaucratic and hierarchical cultures that serve as a barrier to quality improvement implementation. |
| Loper AC/2022 | Not specified | Capacity building | Not specified | Systematic review | Not interventional: Identify principles of capacity building | Both model and tool are not mentioned. | Five essential principles: rigorous, data- and improvement-driven, collaborative, impact-focused, and responsive, and has five core components are facilitate shared learning; data use and improvement; assessment, improvement, and evaluation; communicate and support feedback loops; and cultivate a culture of CQI |
| Swanson TK/1994 | Not specified | Acceptability | Not specified | Quantitative | Not interventional: evaluate the attitudes toward and acceptance of CQI by a family practice residency program's providers and staff after 3 years' experience with the process | Both model and tool are not mentioned. | Faculty, resident, and staff members accepted the CQI process and perceived to be a useful tool in clinical practice: 88% of the faculty, 64% of the residents, and 82% of the staff believed CQI to be useful tool in healthcare clinic |
| Dadi TL et al 2023 | Ethiopia | Services availability and utilization | Impact of CQI | Quantitative | QI interventions: PDSA cycle: continuous planning, implementation, evaluation and handover to local organizations. Planning phase: 1. situational analysis, 2. baseline assessment, 3. set objectives and logic framework, 4. design intervention plan; Implementation Phase: 1. provision of medical equipment & supplies, 2. Capacity building training on RMNCH standards, communication skills, problem-solving and teamwork, 3. Establish QI team, 4 QI coaching, mentoring and supportive supervision, 5. Reward for better performing health facilities annually, and 6. Collaborative learning and experience sharing; Performance assessment and monitoring: Quarterly review meetings, baseline, midline and endline assessment and root cause analysis; Handover and sustainability: provided recommendations and lessons learnt to the woreda health offices, regional health bureau and the health facility, and national ministers and stakeholders. | PDSA | The QI intervention increased the quality of antenatal care by 29.3%, correct partograph use by 51.7% and correct active third-stage labour management, a 19.6% improvement from the baseline, but not significantly associated with improvement in contraceptive service uptake. |

FOCUS-PDCA: find, organize, clarify, uncover, start, plan, do, check and act)
